# A systematic investigation of human DNA preservation in medieval skeletons

**DOI:** 10.1038/s41598-020-75163-w

**Published:** 2020-10-26

**Authors:** Cody Parker, Adam B. Rohrlach, Susanne Friederich, Sarah Nagel, Matthias Meyer, Johannes Krause, Kirsten I. Bos, Wolfgang Haak

**Affiliations:** 1 Max Planck Institute for the Science of Human History, Jena, Germany; 2 ARC Centre of Excellence for Mathematical and Statistical Frontiers, The University of Adelaide, Adelaide, SA Australia; 3 Landesamt für Denkmalpflege und Archäologie, Sachsen-Anhalt, Halle (Saale), Germany; 4 Max Planck Institute for Evolutionary Anthropology, Leipzig, Germany

**Keywords:** Population genetics, High-throughput screening, Biological anthropology

## Abstract

Ancient DNA (aDNA) analyses necessitate the destructive sampling of archaeological material. Currently, the cochlea, part of the osseous inner ear located inside the petrous pyramid, is the most sought after skeletal element for molecular analyses of ancient humans as it has been shown to yield high amounts of endogenous DNA. However, destructive sampling of the petrous pyramid may not always be possible, particularly in cases where preservation of skeletal morphology is of top priority. To investigate alternatives, we present a survey of human aDNA preservation for each of ten skeletal elements in a skeletal collection from Medieval Germany. Through comparison of human DNA content and quality we confirm best performance of the petrous pyramid and identify seven additional sampling locations across four skeletal elements that yield adequate aDNA for most applications in human palaeogenetics. Our study provides a better perspective on DNA preservation across the human skeleton and takes a further step toward the more responsible use of ancient materials in human aDNA studies.

## Introduction

The study of ancient DNA (aDNA) has progressed rapidly over the past decade following the introduction of next generation sequencing^1–3^, where genome-level analyses of archaeological specimens are now standard^4–12^. The increased analytical resolution offered by large scale datasets, coupled with the establishment of laboratory techniques that permit parallel processing of large sample sizes, has resulted in an increasing demand for ancient skeletal samples for assessment of human population genetics, microbiome ecology, and investigations of pathogen evolution. Laboratory processing of ancient remains is intrinsically a destructive process^13–16^, which poses ethical challenges related to the use of irreplaceable resources. Coupled with the high processing costs of aDNA work (from the perspective of both financial and time investments), there is benefit in optimizing approaches for material sampling. Multiple investigations have demonstrated superior human aDNA preservation in the dense inner petrous pyramid, the portion of the temporal bone that houses the inner ear. This observation is based on a collection of comparative PCR^15,17–20^ and whole genome aDNA surveys^16–18,21,22^ that were often limited in either the number of individuals and/or skeletal elements tested. Despite the paucity of a systematic comparative analysis of preservation across the skeleton, aDNA obtained from the petrous portions of human remains has been utilized to great success in the contexts of both ancient human population genetics (e.g*.*^23–27^) and forensic investigations^17,28^.

Historically, sampling of the isolated petrous pyramid has typically involved sectioning or sand-blasting of the temporal bone to isolate the cochlea^16^, making this a highly destructive process^13^. Recent advances in minimally invasive sampling techniques^29^ have led to a better balance between preservation of the anthropological record and the need for the production of reliable genetic data^30,31^; however, the threat of damage to internal microstructures that form an important basis of morphological assessments^32–34^ can still introduce hesitancy on the part of curators and physical anthropologists in making the petrous pyramid available for aDNA applications. These factors, in conjunction with the chance of incomplete recovery of crania at excavation^35^ or restricted sampling of highly valued skeletal samples, make the identification of alternative sampling locations based on quantitative evaluations of DNA preservation across the skeleton of clear benefit. Teeth have been widely used for the study of aDNA^36,37^, though the 30-fold covered genome of an archaic hominin from Denisova Cave from a distal phalanx demonstrates molecular preservation in elements that are not typically considered for paleogenetics work^4^. Despite these successes, a systematic and extensive study of differential DNA preservation across multiple human skeletal elements, such as those done in the context of modern forensics^38,39^, has yet to be attempted on archaeological remains. Our limited understanding of DNA preservation across the human skeleton is a significant hurdle for the efficient, practical, and ethical study of aDNA, which has particular relevance to the field of ancient population genetics where large sample sizes are needed for robust analytical resolution.

**Figure 1:**
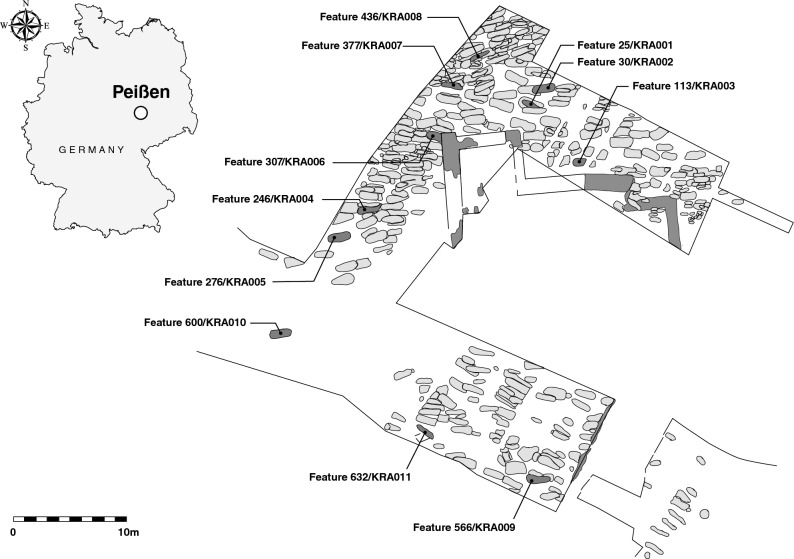
Map of the Krakauer Berg excavation. Graves corresponding to individuals sampled are denoted with both the archaeological ID and assigned sample name.

DNA preservation can be influenced by many factors. Chronological age shows some relationship with the deamination of terminal bases, though this has been demonstrated to play a secondary role to other factors such as environmental and climatic conditions contributing to the overall thermal age of a sample^40,41^. Additionally, burial practises, post-mortem treatment of the deceased, and geology may also influence DNA survival^42^. Beyond these historical factors affecting DNA preservation, laboratory processing methods, such as bleach pre-treatments to remove contamination (e.g.^43–45^), may also affect DNA recovery from a sample. To serve as a baseline for future investigations seeking to incorporate and extrapolate the effects of these sources of variation, e.g. across other species, time series, or geographic regions we present a broad survey of aDNA preservation across a range of skeletal elements. Our source material, consequently, has been deliberately restricted to one archaeological site and time period to control for these factors that can influence molecular recovery as much as possible. The range of elements chosen for this survey consist of petrous bones (chosen for their demonstrated value in aDNA recovery^21,22^), in situ molars, clavicles, the first ribs, thoracic vertebrae, metacarpals, distal phalanges, ischial tuberosities, femora (once widely used in ancient DNA studies^46^), and tali. Multiple locations on each element were sampled from and evaluated for DNA content. A detailed list of skeletal elements, sampling locations and the rationale for why each element was selected for study is provided in Table 1 (see also Supplementary Material: Section 1.2; additionally, for discussion of the sampling of epiphyseal plates, which were not present in sufficient numbers for statistical analyses, see Supplementary Material: Section 2.5). Differential DNA preservation across these elements was investigated in individuals excavated from the church cemetery associated with the abandoned medieval settlement of Krakauer Berg, near Peißen, Saxony-Anhalt, Germany (Fig. 1). Overall, the site exhibited excellent morphological preservation, as expected from a medieval burial in a temperate region. Though preservation of this scale is often not observed in older material or that obtained from climatic regions less suited to molecular preservation, the sampling from complete (or nearly complete) skeletons was a prerequisite for this study in order to maximize the comparability of the elements selected for analyses while also maximizing the chances of successful DNA extraction from each sampling location. It should be noted that the findings of this study are presented as a first systematic exploration of human DNA preservation within a single temporal and geographic context. Whether the trends we report will scale globally can only be determined via similar undertakings of material that derives from different preservation contexts.

**Table 1:** Sampling locations across all skeletal elements and the rationale behind each.

Skeletal element	Rationale	Sampling location	Rationale
Molar (n = 11)	Widely used in aDNA studies and easily available, in situ molars preferentially selected for best preservation	Cementum	Previously shown to be an excellent source of ancient human aDNA^21^
		Dentin	Frequently used in aDNA studies^14,15,36,55^
		Pulp	Preferred option in pathogen (i.e. *Yersinia pestis*) studies^34,67,70^
Petrous pyramid (n = 11)	Currently most sought-after skeletal element for aDNA research	Dense cochlear portion	Currently considered the best source of endogenous ancient human DNA^21,22^
Clavicle (n = 10)	Highly vascularized tissue, not studied in terms of aDNA retention	Cortical bone from shaft	Cortical bone previously shown to harbour the most endogenous human aDNA^36,48^
		Cancellous bone from facet	Richly vascularized^a^
Rib (n = 11)	Readily available	Cortical bone from shaft	Cortical bone previously shown to harbour the most endogenous human aDNA^36,48^
		Cancellous bone from facet	Richly vascularized^a^
Thoracic vertebrae (n = 11)	Readily available	Cortical bone from spinous process	Cortical bone previously shown to harbour the most endogenous human aDNA^36,48^
		Cortical bone from vertebral body	Cortical bone previously shown to harbour the most endogenous human aDNA^36,48^
		Cancellous bone from vertebral body	Richly vascularized^a^
		Cortical bone from neural foramen	Cortical bone previously shown to harbour the most endogenous human aDNA^36,48^
		Cortical bone from superior vertebral arch	Cortical bone previously shown to harbour the most endogenous human aDNA^36,48^
Metacarpal (n = 11)	Readily available	Cortical bone from shaft	Cortical bone previously shown to harbour the most endogenous human aDNA^36,48^
		Cancellous bone from head	Richly vascularized^a^
Distal phalanx (n = 10)	Shown previously to be a good source of ancient human DNA^4^	Cortical bone from pad	Cortical bone previously shown to harbour the most endogenous human aDNA^36,48^
		Cancellous bone from head	Richly vascularized^a^
Ischial tuberosity (n = 9)	Dense, weight bearing bone not studied previously for aDNA retention	Cortical bone from exterior surface	Cortical bone previously shown to harbour the most endogenous human aDNA^36,48^
		Cancellous bone from interior	Richly vascularized^a^
Femur (n = 11)	Long-bone commonly used in the early aDNA studies^46^	Cortical bone from shaft	Cortical bone previously shown to harbour the most endogenous human aDNA^36,48^
		Cancellous bone from head	Richly vascularized^a^
Talus (n = 10)	Dense, weight bearing bone, not studied previously for aDNA retention	Cortical bone and compacted cancellous bone from exterior surface	Consists primarily of densely compacted trabecula with a very thin coating of cortical bone
		Cancellous bone from interior	Richly vascularized^a^

^a^Vascularization has been theorized to effect the recovery of pathogen DNA from ancient remains^37,69^, and as such was used as a selection criteria in order to assess if there is a similar effect on host DNA preservation.

To our knowledge, this study presents the most comprehensive systematic evaluation of aDNA preservation across the human skeleton in the published literature. While we further confirm the superior performance of cortical bone stemming from the cochlear portion of the petrous pyramid to yield the highest amounts of recoverable human DNA^21,22,47^, several alternative sampling locations are identified as suitable for downstream population genetic analyses such as the tali, distal phalanges, vertebrae, and teeth.

## Results

Our analytical matrix consists of shotgun sequencing data from single-stranded DNA libraries stemming from 23 separate sampling locations paired end sequenced (2 × 75 cycles) to approximately 5,000,000,000 reads each (Table 1; Supplementary File 1: Source; Raw reads sequenced). These were obtained from ten skeletal elements from each of eleven individuals who were all buried, excavated, documented, stored, sampled from, and ultimately processed and sequenced under the same conditions, in order to eliminate as many confounding variables as possible. All individuals selected for study had at least nine elements available, and all elements were present in at least nine individuals. In total, this resulted in 246 single-stranded aDNA libraries for comparison. In addition, as the use of hybridization capture technology in aDNA studies has become a popular alternative to shotgun sequencing^27^, an additional 87 libraries were subsequently enriched by hybridization capture for 1,240,000 informative variant SNPs across the human genome using the 1240k^27^ human SNP array and paired end sequenced (2 × 75 cycles) to a depth of approximately 40 million reads each. Our goals in evaluating this dataset are to ascertain which of the chosen sampling locations are most efficient in terms of authentic host DNA recovery, processing cost, and limiting damage to the anthropological record. To achieve a balance between aDNA recovery and drilling damage, as well as to more accurately compare the expected yields from a single instance of sampling, each sampling location was screened only one time. Additionally, all skeletal remains were sampled from approximately the same location on each bone by the same worker (CP) in order to minimize differences in DNA yield and quality stemming from the natural variations in DNA preservation within each individual skeletal element and the effect of inter-observer variations in sampling procedures (i.e. potential variations from one area of a bone to the next^48^). Analyses normalized in terms of input material available from each sampling location can be found in the provided Supplementary text in Section 2.4. The results for each metric examined in this study are presented in the chronological order in which they are typically assessed, in the experience of the research team, and are not prioritized in any subjective order of importance.

**Figure 2:**
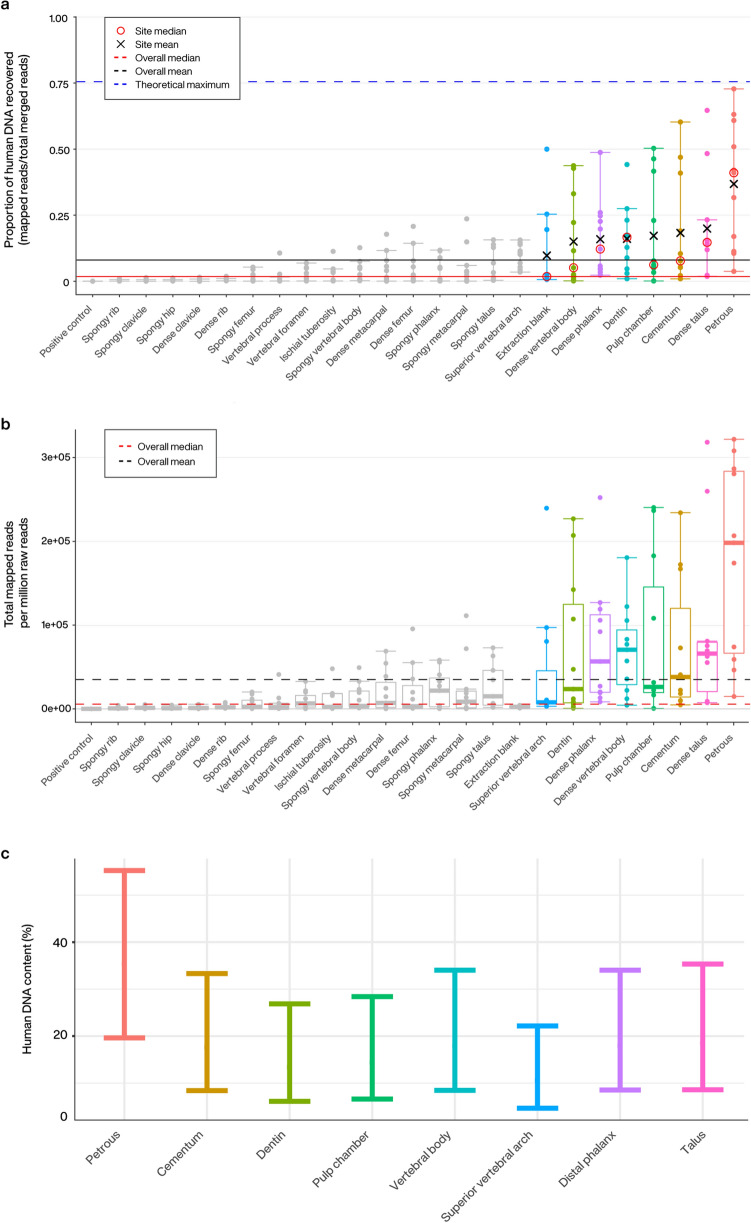
(**a**–**c**) Human DNA content for all screened samples. Black lines represent the overall mean, red the median (solid: human DNA proportion, dashed: mapped human reads per million reads generated). Individual sampling locations with an average human DNA proportion higher than the overall mean (8.16%) are colourized in all analyses. (**a**) Proportion of reads mapping to the hg19 reference genome. The blue dashed line represents the theoretical maximum given the pipeline’s mapping parameters (generated using Gargammel^94^ to simulate a random distribution of 5,000,000 reads from the hg19 reference genome with simulated damage). Individual means (black X) and medians (red circle) are reported for those samples sites with a higher average human DNA proportion than the overall mean. (**b**) Number of unique reads mapping to the hg19 reference genome per million reads of sequencing effort (75 bp paired end Illumina). (**c**) Predicted range of expected human DNA recovery (in proportion of total reads) for each top scoring sampling site. Predictions were generated using a beta-fitted mixed effects model to simulate 55,000 sampling iterations.

One of the most frequently used metrics for the evaluation of successful DNA recovery in human archaeological material is the proportion of human DNA recovered relative to DNA from other sources. This is often the first criterion considered to determine if a sample is suitable (both economically and analytically) for further testing. In this context we examined the average proportion of total (prior to duplicate removal) human DNA recovered post paired-end read merging, accommodating filters for sequence length and mapping quality (see “Methods”: Eq. 1). Among the 23 sampling locations we find the highest average proportion of human DNA in the petrous pyramid (34.70% human DNA on average), followed by dense tissue obtained from the neck and articular surfaces of the talus (21.25%), the cementum (18.97%), cortical bone from the distal phalanx (18.89%), material from the dental pulp chamber (15.09%), cortical bone from the vertebral body (15.04%), the dentin (14.27%), and cortical bone from the superior vertebral arch (8.32%). All other sampling locations evaluated contained an average human DNA proportion lower than the overall average of 8.16% (Fig. 2a, Supplementary File 1: % mapping q37) across all elements tested.

To provide a realistic approximation of the cost efficiency of human DNA retrieval from each sampling location and to estimate complexity within each single-stranded library, we further compared the average number of unique human reads per million reads of sequencing effort across all samples (see “Methods”: Eq. 2). Here we again find the highest average in the petrous pyramid (1.14 × 10^5^ unique reads mapping per million), followed by the talus (6.43 × 10^4^ unique reads mapping per million), the dental pulp chamber (5.26 × 10^4^ unique reads mapping per million), the distal phalanx (5.23 × 10^4^ unique reads mapping per million), cementum (4.89 × 10^4^ unique reads mapping per million), the vertebral body (4.81 × 10^4^ unique reads mapping per million), the dentin (4.76 × 10^4^ unique reads mapping per million), and the superior vertebral arch (2.79 × 10^4^ unique reads mapping per million). All other sampling locations fall below the overall average of 2.43 × 10^4^ (Fig. 2b, Supplementary File 1: Unique reads/million reads). Furthermore, an average unique reads/million lower than that found in the highest of our extraction blanks (2.96 × 10^3^ unique reads per million) was observed in all sampling locations on the ribs and clavicula, as well as cancellous material from the ischial tuberosities. When normalized to reflect the amount of input material from each sampling effort, we find those sampling locations with the lowest available input material to yield the highest average number of unique mapping reads per million per mg of input material, followed by the petrous pyramid (cementum: 3751 unique reads mapping/million reads/mg, material from the pulp chamber: 2736 unique reads mapping/million reads/mg, and petrous pyramid 2087 unique reads mapping/million reads/mg) (Supplementary Figure S14, Supplementary File 1: Unique reads/mg/million), suggesting that material from the cementum and dental pulp chamber may be especially rich in human DNA.

While human DNA content in the negative controls was relatively high on average (10.77%), this metric is not directly informative for the evaluation of potential contamination as there are comparatively few DNA molecules in negative controls and as a result high numbers of amplification rounds are typically required, yielding an abundance of clonal PCR duplicates (see Supplementary File 1: Reads raw sequencing effort, Reads after merging, and Unique reads/million reads). The number of unique mapping reads per million is, therefore, a more informative metric.

Here the average among our controls is an order of magnitude lower than what we report for our samples (an average of 1.67 × 10^3^ unique reads mapping per million in extraction blanks vs an average of 2.43 × 10^4^ unique reads mapping per million reads overall; see Supplementary File 1: Unique reads/million reads). Using this approach, we considered all individual sampling efforts that yielded a lower number of unique reads/million than what was observed in the highest of the negative controls (2.96 × 10^3^ unique reads mapping/million reads) to be unsuccessful, regardless of potential authenticity as determined by characteristic patterns of DNA decay typically indicating ancient origin. (see Supplementary File 1: Damage signals). With this in mind, however, all “failing” samples were retained for all downstream comparative analyses so as to more accurately represent the expected outcomes of sampling efforts across a given sampling location. We additionally observed that all cancellous samples, as well as cortical bone samples stemming from ribs, claviculae, metacarpals, ischial tuberosities, femora, neural foramen and spinous process of the thoracic vertebrae (15 sampling locations, n = 158) exhibited average human DNA contents lower than the overall averages (> 8.16% for human DNA proportion, and 2.43 × 10^4^ for unique human reads/million reads) making them unlikely to be among the most efficient sampling locations in any metric. Accordingly, we removed these sampling locations from further analyses to allow for the deeper investigation of the remaining eight sampling locations consisting of the dentin, cementum, and dental pulp chambers as well as cortical bone from the cochlear portion of the petrous pyramid, vertebral body, superior vertebral arch, distal phalanx, and talus (eight sampling locations, n = 87).

Restriction of our dataset to these eight sampling locations also permitted generation of a predictive model of expected human DNA yields via mixed effects beta regression (Fig. 2c). Using this approach, we were able to account for unavoidable sources of variation such as those stemming from individual preservation at particular skeletal locations (i.e. the natural variability among sampling locations across individuals). Due to the high variability of the proportion of human DNA recovered across both sampling locations and individuals, 55,000 iterations of this simulation were run to evaluate overall consistency of the expected proportion of human DNA recovered from each sampling location (Supplemental Material: Table S1). Here, the petrous pyramid significantly outperformed all other tested elements in terms of the expected range of proportions of recovered human DNA (all p-values < 0.0279), and yielded the highest predicted proportion of human DNA in the greatest number of simulations (41.87% of 55,000 simulations). The seven remaining alternative sampling locations on four other elements, although second to the petrous pyramid, also exhibited excellent human DNA recovery with yields statistically indistinguishable from each other (p-values > 0.1) (Fig. 2c). The distal phalanx, vertebral body, cementum and talus yielded the highest proportion of human DNA in 9.93–10.61% of simulations, followed by the pulp chamber, dentin, and superior vertebral arch, which yielded the highest proportions in 4.28–7.22% of the simulations.

**Figure 3:**
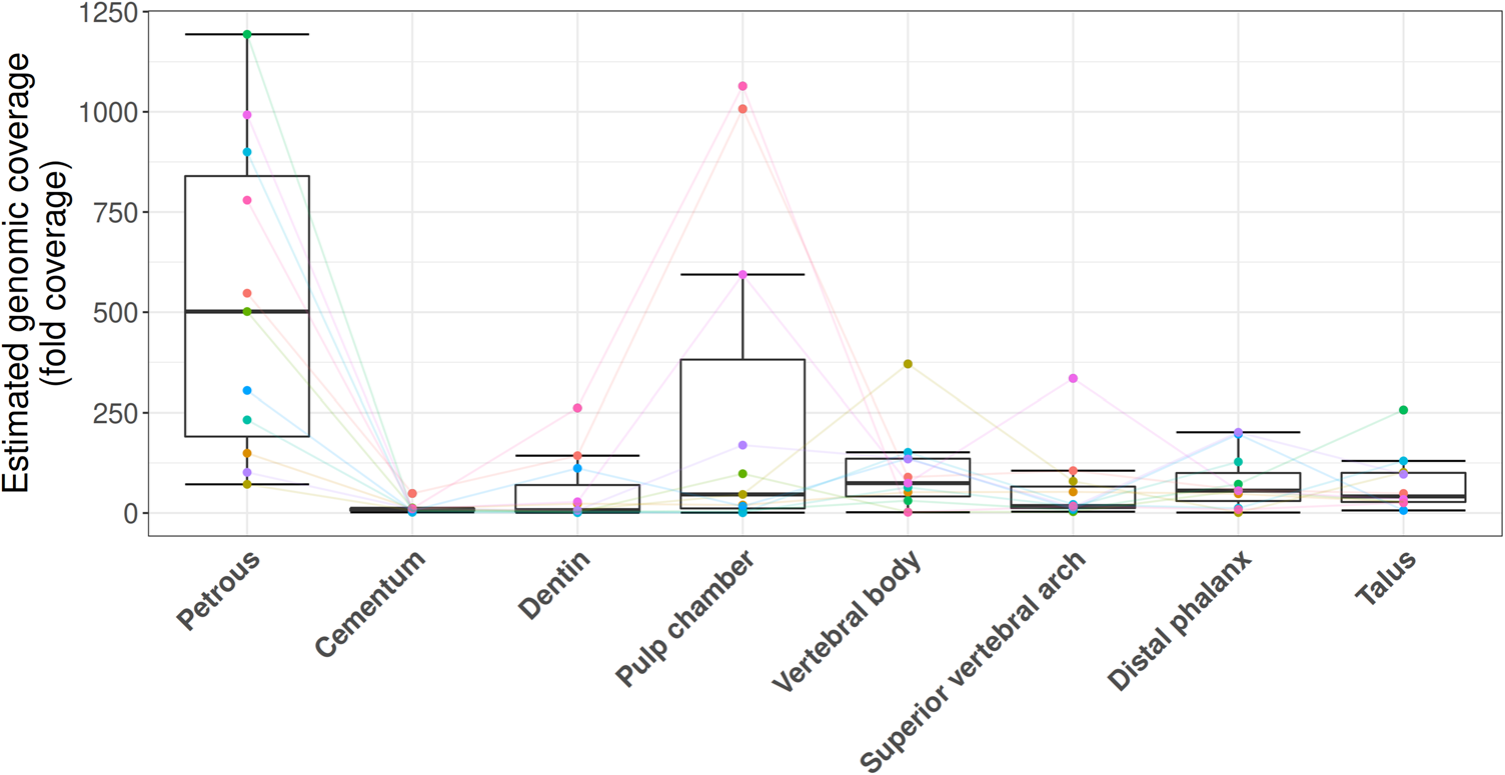
Estimated fold coverage of the hg19 reference genome contained within each single-stranded library. Coloured points and lines denote sampling across individuals.

Although the proportion of human DNA is vitally important for the identification of suitable sampling locations, both the quantity and quality of that DNA are also important for the success of downstream analyses. With that in mind, we examined several additional aspects of DNA preservation. As many studies require the confident assignment of genetic variants at individual loci, it is important that aDNA libraries are of sufficient complexity and show low signals of contamination with present-day human DNA. The aDNA libraries produced in this study were not sequenced to exhaustion, and as a consequence duplication rates were too low to be informative in terms of estimating library complexity in both the pre-enrichment libraries (average duplication factor 1.21) and the post-capture libraries (average duplication factor 1.22) (see Supplementary File 1: Duplication factor). Instead, we used the number of unique molecules in each library as determined by quantitative PCR and the proportion of mapped sequences to estimate the total genomic coverage within each library^49^ as a predictor of library complexity (see “Methods”: Eq. 3). The range of estimated genomic coverages within each sampling location was asymmetrically distributed and the data were subsequently transformed by a factor of X^0.1^ in order to fit a linear model, as suggested by Box–Cox transformation, to evaluate significance (untransformed data is shown in Fig. 3 and Supplementary file 1: Est. genomic coverage; for transformed analyses see Supplementary Figure S11). Here, the petrous pyramid has the greatest potential to provide higher genomic coverage from an individual library (untransformed median estimated genomic coverage 501.55×, p-values < 0.0056), where all other sampling locations aside from the cementum were statistically indistinguishable (untransformed median estimated genomic coverages for each sampling location: 74.54× for the vertebral body, 55.94× for the phalanx, 46.51× for the pulp chamber, 41.44× for the talus, 17.38× for the superior vertebral arch, and 7.14× for dentin). DNA libraries derived from cementum yielded significantly lower estimates of genomic coverage within each library compared to all other sampling locations (untransformed median of 10.42×, p-values < 0.047) except for those libraries from dentin and the superior vertebral arch (Fig. 3). Normalized for input material, cementum yielded slightly higher median genomic coverage than that observed in the dentin and superior vertebral arch (0.46×, 0.16×, and 0.31× per mg input respectively) while the petrous pyramid yielded the highest (7.96× per mg input), followed by material from the pulp chamber (2.14× per mg input) (see Supplementary Figure S15, Supplementary File 1: Est. genomic coverage/mg).

**Figure 4:**
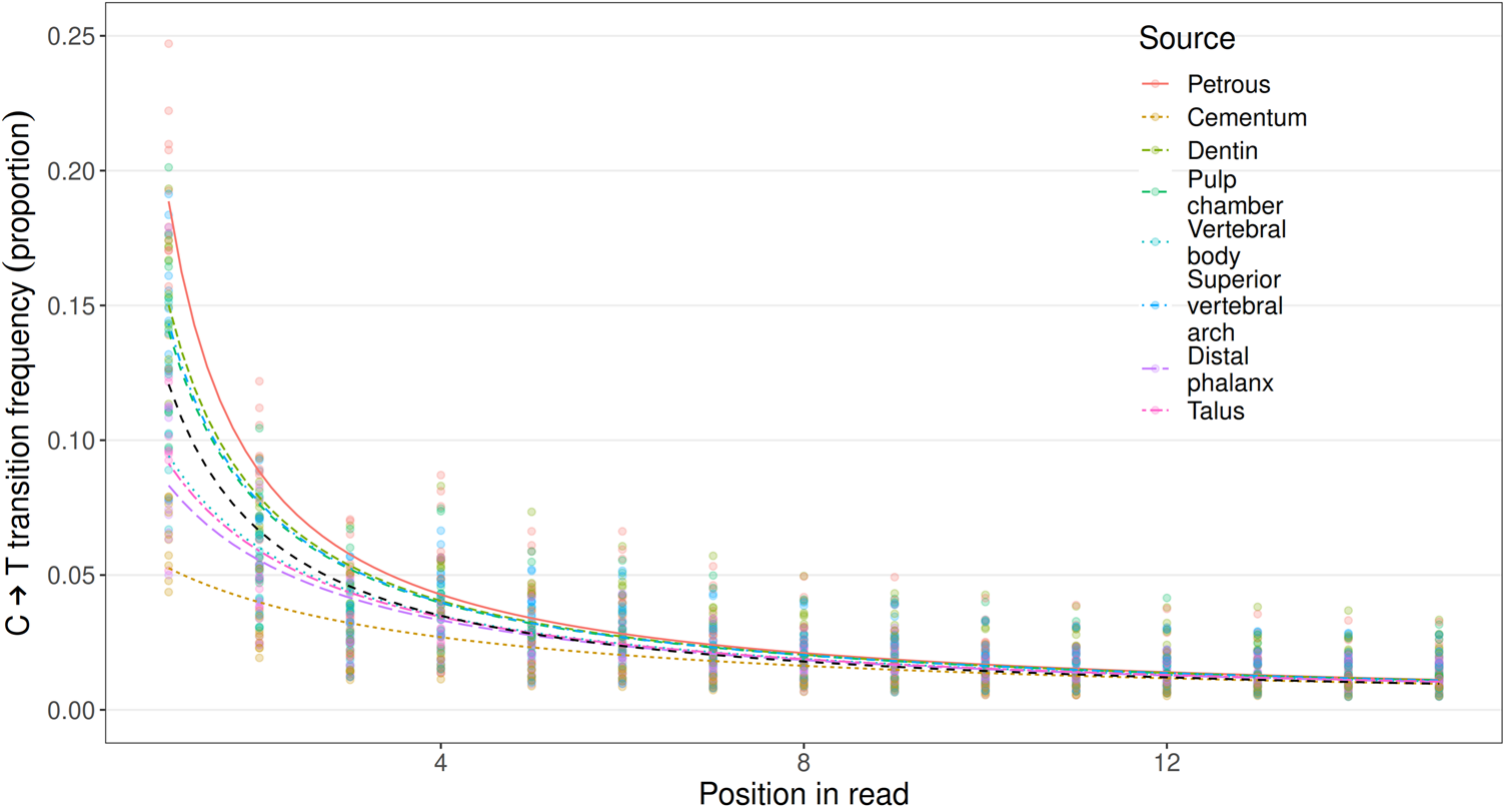
Average proportion of C → T transitions as observed in the first 15 reads of the 5′ end of reads. The black line represents the mean damage observed across all elements and individuals. Coloured lines indicate the average proportion of transitions within sampling locations, while points represent the corresponding range of individual data within each sampling location.

Additionally, we find significant variation in both the frequency of C → T damage caused by nucleotide misincorporations at the ends of the reads and how far into the reads this signal can be detected (Fig. 4, Supplementary File 1: Damage signals). Within sampling locations, variations in the frequency of C → T damage patterns were very low (Supplementary Figure: S13, Supplementary File 1: Damage signals), suggesting that the observed variations across sampling locations are unlikely to result from modern human contamination. Reads generated from the petrous pyramid have the highest damage signal, a 5′ terminal C → T frequency of approximately 21% on average (all pairwise comparison p-values < 0.001). By comparison, cementum shows significantly lower signals than all other sampling locations (all pairwise comparison p-values < 0.001), with approximately half this frequency of damage at the terminal 5′ position. The distal phalanx, talus, and vertebral body form a statistically indistinguishable group with deamination frequencies slightly higher on average compared to the cementum, followed by the dentin, the dental pulp chamber, and the superior vertebral notch, with deamination frequencies lower than the petrous pyramid but higher than the aforementioned group (all pairwise comparison p-values between groupings < 0.001).

Contamination estimates based on X chromosome mapping coverage were calculated for all enriched libraries originating from individuals genetically assigned as male (n = 7, 8 samples per individual, 56 total samples) using the ANGSD pipeline^50^ to scan known informative SNPs on the X chromosome for polymorphisms. All but one of the 56 samples exhibited low contamination with values statistically indistinguishable across sampling locations (< 4% X chromosome contamination for all enriched libraries from all sampling locations other than the superior vertebral arch of individual five (KRA005), which exhibited contamination levels of 19.52%; p-value = 0.48; see Table 2; also see Supplementary Materials Section 2.6: Additional measures of contamination and discussion of mitochondrial contamination estimates^51,52^).

**Table 2:** Duplication levels, average fragment length, and X chromosome contamination estimates for top performing sampling locations.

Sampling location	Average cluster factor (#all mapping reads/#unique reads) pre-enrichment (post-enrichment)	Average fragment length (median) in bp	Contamination estimates (X chromosome; average proportion of human DNA)	Average number of SNPs covered on X at ≥ 3× (per million reads)
Petrous pyramid	1.188 (1.159)	65.40 (60.09)	0	73.83
Cementum	1.197 (1.288)	67.28 (61.36)	0.011	94.78
Dentin	1.188 (1.283)	60.22 (55.54)	0.002	57.33
Pulp	1.179 (1.206)	55.14 (50.55)	0.013	44.88
Distal phalanx	1.191 (1.257)	65.95 (59.36)	0.013	127.75
Vertebral body	1.194 (1.247)	66.14 (60.54)	0.008	119.71
Superior vertebral arch	1.190 (1.208)	63.02 (57.91)	0.021^a^	51.13
Talus	1.198 (1.206)	68.20 (62.40)	0.011	92.50

^a^The sample from KRA005 was removed as an outlier with a very high (0.195) contamination estimate.

**Figure 5:**
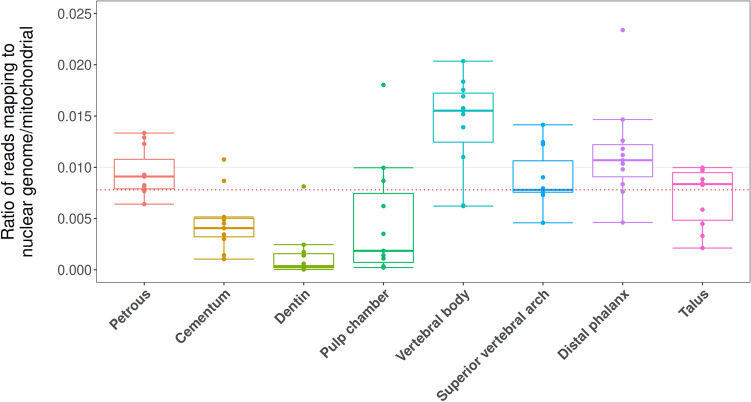
Ratio of reads originating from the nuclear genome to those of the mitochondrial genome. The black line denotes the overall average, the red the overall median.

Average read lengths and the ratio of nuclear genome read recovery to those mapping to the mitochondrial genome (NUC/MT) were also evaluated across the eight sampling locations with the highest average human DNA proportions. After filtering to remove all reads < 30 bp, the dental pulp chamber housed significantly shorter reads in comparison to all other sampling locations except for dentin (averages of approximately 55 bp and 60 bp respectively, pair-wise p-values < 0.019) (Table 2, Supplementary File 1: Average length), with no significant variation observed between any other sampling locations. An asymmetrical distribution of the NUC/MT ratio was observed within sampling locations and as such was transformed by a factor of X^0.5^ to fit our model (for visual analyses of transformed data see Supplementary Figure S12). We find that nuclear reads were lowest in dentin (untransformed median 1:2769, p-values < 0.011), followed by the pulp chamber (untransformed median 1:539 and not significant when compared to cementum, p-value > 0.45), with all other sampling locations statistically indistinguishable (individual untransformed medians 1:64 in the vertebral body, 1:94 for the distal phalanx, 1:109.86 in the petrous pyramid, 1:128 in the superior vertebral arch, and 1:246 in the cementum) (Fig. 5, Supplementary File 1: NUC/MT). Average GC content was calculated for all libraries from the eight sampling locations with average human DNA proportions higher than the mean (8.16%) and ranged between 37.14% and 39.87% (see Supplementary File 1; GC content).

**Figure 6:**
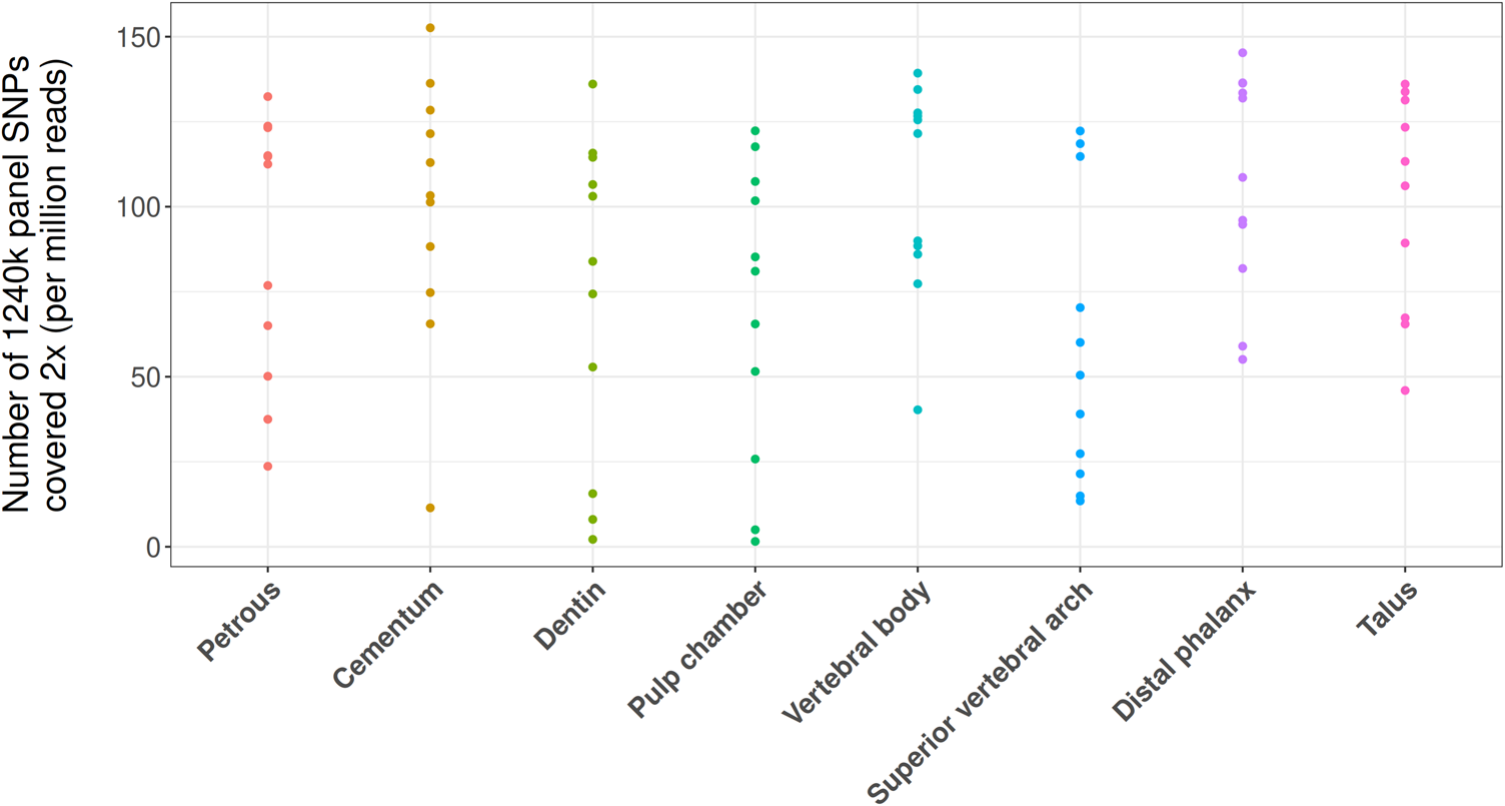
Comparison of 1240k SNP positions covered at least 2× post-capture across skeletal elements normalized by sequencing effort (number of raw reads generated) shown in SNPs per million reads generated.

Since many aDNA analyses, especially those used in population genetics, require a relatively high coverage of informative loci across the genome, libraries are often enriched for these loci by targeted capture. In our case, this was done for the eight sampling locations that yielded human DNA in proportions higher than the calculated mean for our dataset. To determine the practical usability of the data generated, we compared the relative number of SNPs covered by at least two reads (per million reads sequencing depth) post-1240k capture-enrichment across these eight sampling locations. Here we find that SNP coverage per million reads sequencing effort is statistically indistinguishable between sampling locations. Given that these libraries were not sequenced to exhaustion, this strongly suggests all of these sampling locations are equally suited for SNP analyses at our current sequencing depths (Fig. 6). When normalized for available input material the cementum provided significantly higher SNP coverage than all other sampling locations (p-values < 0.02) (see Supplementary Figure S16). As an alternative example of practical usability, we also investigated the phylogenetic resolution for Y-haplotype assignment among all seven male individuals using the ISOGG list of diagnostic SNPs (current as of 26 November 2019) to determine how confidently Y-haplogroups could be called at the approximately 40 million read sequencing depth considered here. The resolution of Y-haplotype assignment was high across most elements and individuals (Table 3). In two individuals (KRA003 and KRA004), the dentin and pulp chamber had a much lower resolution compared to other elements; however, this is most likely an artefact of the low human DNA proportions observed in these samples both before and after SNP capture (Supplementary File 1: % mapping q37, Sheets 1 and 2 respectively), rather than any biological trend.

**Table 3:** Y-haplotyping resolution post-1240k enrichment across all males and associated sampling locations.

Individual	Y-haplogroup resolution (ISOGG SNP positions covered)							
	Petrous	Cementum	Dentin	Pulp	Superior vertebral arch	Vertebral Body	Distal phalanx	Talus
KRA001	R1a1a1b1a1a1c (24,624)	R1a1a1b1a1a1c1 (22,102)	R1a1a1b1a1a1c (20,192)	R1a1a1b1a1a1c (16,052)	R1a1a1b1a1a1c (23,345)	R1a1a1b1a1a1c1 (17,492)	R1a1a1b1a1a1c (8383)	R1a1a1b1a1a1c (11,475)
KRA003	R1a1a (6540)	R1a1a1b1a1a (20,569)	R (1060)	R (2919)	R1a1a1b1a1a1c (22,012)	R1a1a1b1a1a1c1 (28,747)	R1a1 (9689)	R1a1a1 (16,716)
KRA004	I1a2a1a1d (26,477)	I1a2a1a1d (26,305)	N/A^a^ (271)	I1 (7186)	I1 (2682)	I1a2a1a1d (16,032)	I1a2a1a1d (28,127)	I1a2a1a1d (24,327)
KRA005	E1b1b1a1b1a (29,675)	E1b1b1a1b1a (27,699)	E1b1b1a1b1a (14,366)	E1b1b1a1b1a (15,098)	E1b1b1a1b1 (5470)	E1b1b1a1b1a (27,296)	E1b1b1a1b1a (30,390)	E1b1b1a1b1a (33,106)
KRA008	I2a1a2b1a1a (9606)	I2a1a2b1a1 (28,209)	I2a1a2b1a1 (26,795)	I2a1a2b1a1 (17,317)	I2a1a2b1a1 (10,267)	I2a1a2b1a1a (26,993)	I2a1a2b1a1a (28,079)	I2a1a2b1a1 (10,683)
KRA009	R1a1 (4616)	R1a1a1b1a1a (11,042)	R1a1a1b1a1a1c (16,815)	R1a1a1b1a1a1c (16,942)	R1a1a1b1a1a1c1 (12,172)	R1a1a1b1a1a1c1 (23,230)	R1a1a1b1a1a1c (30,160)	R1a1a1b1a1a1c1 (30,787)
KRA010	J2b2a1a1a1a1a (22,337)	J2b2a1a1a1a1a1a (23,201)	J2b2a1a1a1a1a1a (21,564)	J2b2a1a1a1a1a1a (28,040)	J2b2a1a1a1a1a1 (27,044)	J2b2a1a1a1a1a1a (26,140)	J2b2a1a1a1a1a (24,591)	J2b2a1a1a1a1a1a (24,697)

^a^Zero resolution in Y-haplotyping.

## Discussion

Based on previous successes in DNA recovery, the petrous pyramid is currently the most sought-after skeletal element for aDNA analyses^21–27^. Our investigation of multiple skeletal elements further confirms the value of the petrous pyramid in the recovery of ancient human DNA (Fig. 2a–c). We also find that single-stranded aDNA libraries constructed from material retrieved from the cochlear region of the petrous pyramid are higher in complexity (in terms of the estimated genomic coverage within each library) than those stemming from all other tested sampling locations (Fig. 3) in line with previous studies^21,28,29^. Importantly, however, libraries stemming from the petrous pyramid performed comparably to those from all other sampling locations in terms of fragment length, number of reads mapping to the nuclear genome (Table 2, Fig. 5, Supplementary File 1: Avg. length and NUC/MT), X chromosome contamination estimates (the lowest of all sampling locations with an average of 0, though not statistically significant, Table 2), and SNP coverage post-1240k enrichment (Fig. 6). Human DNA fragments recovered from the petrous pyramid show a much higher frequency of cytosine deamination than any other element^21^ (Fig. 4, Supplementary Figure S13, Supplementary File 1: Damage signals), which helps to support their authenticity as ancient^40,53–57^. It should be noted however, that a higher frequency of deamination may necessitate either the production of libraries treated with repair enzymes such as uracil-DNA glycosylase^58^ or the removal of damaged bases by read trimming to improve mapping. These treatments, however, can result in an overall reduction in read length which can translate to a lower coverage of the reference genome as some reads may no longer reach minimum read length thresholds. While the comparatively lower deamination signal identified in the other sampling locations here may result from modern DNA contamination, our data shows no overall correlation between the proportion of human DNA recovered and the proportion of terminal cytosine deamination. Additionally, we do not observe higher amounts of contamination in other sampling sites based on our X chromosome contamination analysis (Table 2), nor do we see significant variation in deamination patterns within sampling locations across individuals (Supplementary Figure S14). However, a high overall fragment length in conjunction with low deamination frequencies (as observed in cementum) may be indicative of contamination with modern human DNA^59^. A previous comparison of deamination patterns in cementum and petrous pyramid yielded a similar differential to what we report here^21^, where cementum exhibited approximately half the frequency of deamination at the 5′ terminus with no indication of modern contamination. Despite its excellent potential for human aDNA recovery, sampling from the petrous pyramid may not always be possible for a variety of reasons including hesitancy on the part of curators in regards to potential damage to the anthropological record, despite the fact that in cases where skulls are fully preserved and sampling of the temporal bone would otherwise be particularly damaging, cranial base drilling techniques have recently been investigated and recommended^29^.

In the remaining skeletal elements where higher than average proportions of human DNA were recovered (> 8.16%), we find that in situ molars are inferred to have a high probability of endogenous DNA recovery across all three separate sampling locations (Fig. 2a–c). Library complexity was high in both the dentin and material from the pulp chamber (Fig. 3), and contamination estimates low (Table 3). Cementum stands out as having both the highest average fragment length (Table 3) and the lowest deamination frequency (Fig. 4) which, as previously noted, may indicate elevated levels of contamination with modern human DNA, despite a low contamination signal observed in X chromosome analyses (Table 2). The dentin and pulp chamber, conversely, returned the shortest average read lengths and were second only to the petrous pyramid in terms of having the highest proportion of detectable deamination damage.

In terms of the ratio reads mapping to the nuclear genome/reads mapping to the mitochondrial genome, we find the dentin to harbour far less nuclear material than any other sampling location (Fig. 5, Supplementary File 1 NUC/MT). In particular, we observe a substantial differential in nuclear to mitochondrial mapping reads between the dentin and material from the dental pulp chamber (average ratios of 1:2769 and 1:539 respectively). It should be noted that these two sampling locations are not actually separate tissue types and instead are only differentiated by their physical location within the same substrate. To explain this observation, it is important to look at the process by which dentin is formed. Starting in from the outer surface (mantle) of the tooth, odontoblasts first secrete a type-1 collagen matrix, which is then mineralized in a process similar to the endochondral ossification of bone. However, odontoblasts, unlike their cognates in skeletal tissue, do not become trapped in the resulting hydroxyapatite matrix. Instead, thin extensions of the cell called odontoblast processes (alternatively Tome’s fibres) remain within the calcified matrix, forming permanent channels throughout the dentin (dentinal tubules) while the rest of the cell, including the nuclear portion, migrates inwards towards the pulp chamber^60^. The bulk of the dentin itself is essentially void of nuclear DNA during life, though organelles such as mitochondria can persist within the odontoblast processes. When the odontoblasts die, however, nuclear DNA can bind to the hydroxyapatite matrix along the wall of the pulp chamber^61–63^. The result is an extreme disparity between the number of nuclear reads recovered from the superficial layer of dentin sampled as part of the pulp chamber and the dentin sampled from deeper within the tooth. As a consequence, pulp chamber sampling is generally more suitable for nuclear studies, whereas the deeper layers of dentin are better suited for mitochondrial investigations.

However, the fact that dental samples harbour three sampling locations that performed well in terms of human DNA content and two in terms of post-1240k-capture-coverage is an indication of their value. Our observation that dentin exhibited the lowest post-enrichment coverage out of the top sampling locations could be due to its lower nuclear read to mitochondrial read ratio and thus has fewer nuclear reads in the library available for capture. Of note, despite drilling from multiple locations, the enamel, which is frequently examined in isotope^64,65^, histological^66^ and morphological^67,68^ studies, often remains entirely undamaged throughout the sampling process, as minimally invasive sampling methods for teeth focused on the avoidance of alterations to enamel structures have long been established^67^. Finally, the two sampling locations most limited in available material (in the context of sampling efforts from a single element) are the cementum and the dental pulp chamber. Both of these sampling locations performed well when directly compared to all other sampling locations (with up to 10× more material available for DNA extraction in some cases, Supplementary File 1) regardless of the amount of material used in extraction. When weight of the sample used for extraction is factored in, however, material from the dental pulp chamber and cementum outperforms all sampling locations other than the petrous pyramid with respect to average number of unique reads mapped per mg of input material (Supplementary Section 2.4). This suggests both sampling materials are particularly rich in DNA content though the complexity of this content in the cementum may not be as high as that found in material from the dental pulp chamber. These factors, combined with the known potential for teeth to harbour oral bacterial and pathogen DNA^37,69–72^, make sampling from molars valuable as an alternative to the petrous pyramid.

Two sampling locations on the thoracic vertebrae, namely the cortical bone collected from the vertebral body and the junction of the lamellae and spinous process (the superior vertebral arch) were found to yield high average proportions of human DNA (Fig. 2a–c, Supplementary File 1: % mapping q37 and Unique reads/million). Additionally, library complexity (Fig. 3, Supplementary File 1: Est. genomic coverage), average fragment length (Table 2, Supplementary File 1: Avg. length), post-capture SNP coverage (Fig. 6), nuclear to mitochondrial read ratio (Fig. 5, Supplementary File 1: NUC/MT), and deamination frequencies (Fig. 4, Supplementary File 1: Damage signals) fell well within the ranges of the other top performing sampling locations (aside from the petrous pyramid). As with teeth, thoracic vertebrae have multiple high-yield sampling sites, are often well preserved, have been shown to harbour traces of ancient pathogens such as tuberculosis^73,74^, and in the absence of pathological changes, are of less value in morphological studies given that they are numerous.

Both the talus and distal phalanx exhibited high human DNA recovery rates (Fig. 2a–c, Supplementary File 1: % mapping q37 and Unique reads/million) and showed high average fragment length (Table 2, Supplementary File 1: Avg. length) and complexity (Fig. 3, Supplementary File 1: Est. genomic coverage), as well as low contamination estimates (Table 2), nuclear-mitochondrial read ratios (Fig. 5, Supplementary File 1: NUC/MT), and deamination frequency at the 5′ terminus (Fig. 4, Supplementary File 1: Damage signals). While both elements have been under-utilised in aDNA investigations to date, the distal phalanx has previously been shown to yield sufficient aDNA to reconstruct a 30-fold genome from a Denisovan specimen^4^.

Among the other sampling locations considered in this survey, those yielding human DNA proportions that are, on average, lower than the overall mean (8.16%) were not considered for further analyses, as our goal was to ascertain the most efficient and cost-effective sampling locations from which to retrieve human DNA. As such, we determined that samples from the femur, metacarpal, ischial tuberosity, metacarpal, ribs, and *clavicula,* as well as any samples derived from cancellous (spongy) material (in order of decreasing yield) are all unlikely to yield high amounts of endogenous human DNA. In light of this, we feel sampling from these elements, or from cancellous tissue in general, for aDNA analysis should be avoided if possible to circumvent the needless destruction of archaeological samples for minimal gains.

## Conclusions

As intensifying ethical scrutiny surrounds the field of aDNA with regards to the destruction of irreplaceable archaeological human remains^30,42,75–77^, it is imperative for those conducting such research to maximize the chances of successful data generation from minimally invasive sampling. It is of similar importance to both maximize the potential amount of information obtained from and to simultaneously minimize laboratory processing times for each sampling effort to balance the high cost of aDNA research with the aforementioned ethical considerations. As such, our large cross-sectional evaluation of aDNA recovery across the skeleton helps to facilitate this balance by increasing perspectives on molecular preservation not only in previously studied sampling locations, but also in a set of new ones. Our results demonstrate that, from the locations we consider here, the dense cochlear portion of the petrous pyramid remains the best sampling location for high-quality ancient DNA while sampling from cancellous tissue from any tested skeletal element should be avoided if possible. However, we also report on seven additional sampling locations on four other skeletal elements, all of which performed equally well in relation to each other in our evaluations. Though lesser in respect to proportion of human DNA recovered and library complexity than that observed in the petrous pyramid, these seven sampling locations show promise as suitable alternatives. While our sample set is limited both temporally and geographically, our results are likely informative for other climatic regions, time periods and perhaps even in anatomically comparable species as has already been demonstrated for the petrous portions itself^78–81^. It should also be noted that, as this study has focused on identifying the most efficient sampling locations from which host (in this case human) DNA can be recovered, the sampling strategies and suggestions put forth here may not be applicable in studies seeking to retrieve DNA from pathogens, the microbiome, or other co-cohabitating organisms within the host.

By providing researchers with more varied options for the successful recovery of endogenous ancient human DNA, we hope to provide a framework in which successful collaborations between archaeologists and geneticists can continue to enrich our knowledge of history and heritage. At the same time, continuing efforts to fully optimize our sampling strategies will allow the above collaborations to go forward in a more ethical fashion by minimizing damage to the finite archaeological record.

## Methods

### Sample selection, pre-treatment, and bone powder generation

Individuals from the Krakauer Berg collection housed at the State Office for Heritage Management and Archaeology, Saxony-Anhalt [State Museum of Prehistory, Halle (Saale)] (Fig. 1) were sampled for DNA extraction. This collection consists of approximately 800 individuals and represents a typical medieval burial, with age and sex distribution consistent with an attritional context. Ten skeletal elements were selected as targets for aDNA sampling (Table 1, Supplementary Material: Section 1.2). For each individual, morphological preservation of these pre-selected elements was assessed, and individuals were included in the study if a minimum of eight elements were present and were sufficiently well preserved. This resulted in a study set of eleven individuals, seven males and four females (genetically assigned, see below), who ranged in age at death from approximately 10–45 years, with two juveniles and nine adults. Radiocarbon dating of ribs from each individual (performed at the Curt Engelhorn Centre for Archaeometry in Mannheim, Germany) placed the skeletal series in a time interval of approximately 1040–1402 cal AD (Table 4).

**Table 4:** Biological sex (genetically determined), age at death (archaeologically determined), and calibrated ^14^C dates (in calendar years AD) of individuals selected for aDNA sampling.

Individual (laboratory ID)	Archaeological ID (burial Nr.-individual Nr.)	Sex	Age at death	^14^C dates (AD, Cal 2-sigma)
KRA001	25-1a	Male	25–35	1058–1219
KRA002	20-2a	Female	20–22	1227–1283
KRA003	113-6a	Male	25	1059–1223
KRA004	246-1a	Male	15	1284–1392
KRA005	276-2a	Male	10–12	1170–1258
KRA006	307-4a	Female	30–40	1218–1266
KRA007	377-6a	Female	25–30	1167–1251
KRA008	436-6a	Male	20	1301–1402
KRA009	566-3a	Male	Unknown adult	1158–1254
KRA010	600-7a	Male	25	1276–1383
KRA011	632-2a	Female	30–45	1040–1159

To reduce external contamination as much as possible, all elements were processed in a dedicated ancient DNA laboratory under controlled conditions. Similarly, variation in both skeletal sampling^48^ and DNA extraction was eliminated as much as feasibly possible by allocating these tasks to a single individual (CP). At least two sampling locations (Table 1, Supplementary Material: Section 1.2) were selected for each element other than the petrous pyramid, one of which was comprised of cortical bone and the other of cancellous bone. Sampling of the petrous pyramid followed previously established sampling procedures^47^ and involved the sectioning of the petrous pyramid to allow access to the dense bone surrounding the cochlea for drilling. Sampling of teeth was performed in a three-step process and involved removal of the cementum followed by sectioning and drilling of the pulp chamber and dentin portions. Prior to sampling, all relevant locations on each element were cleaned with bleach (0.01% v/v) via 5-min incubation, followed by rinsing with distilled water and exposure to UV light for 30 min to cross-link any residual surface contamination from modern DNA. Where applicable the outermost surface of bone was removed by abrasion with a standard dental drill (KaVo K-POWERgrip EWL 4941) and size 016 round bit (NTI Kahla). Approximately 100 mg of bone powder was drilled from each sampling location with exception of the cementum and dental pulp chambers where an average of approximately 19 mg (standard deviation of 10.8 mg) and approximately 24 mg (standard deviation of 15.03 mg), respectively, of bone powder was recovered, the entirety of which was used for DNA extraction. An average of approximately 54 mg (standard deviation of 11 mg) of bone powder was used in downstream DNA extractions for all other sampling locations (Supplementary File 1: mg input). For molars, cementum was removed by abrasion using a diamond coated rotary cutting disc (NTI Kahla). The tooth was then sectioned at the cemento-enamel junction using a jeweller’s saw (Präzisions-Sägebogen Antilope, with 75 mm blade). Powder from a first pass drilling of the pulp chamber was collected before further sampling of the underlying dentin (Supplementary Material: Section 1.2).

### DNA extraction, library preparation, and sequencing

All DNA extractions were conducted in the clean room facility of the Department of Archaeogenetic of the Max Planck Institute for the Science of Human History (MPI-SHH) located in Jena, Germany, using a modified filter column protocol^14^ (Supplementary Section 1.3.1). Single-stranded DNA libraries^82^ were prepared from all extracts by automation^83^ using the Agilent Bravo Liquid Handling System at the Max Planck Institute for Evolutionary Anthropology in Leipzig, Germany. Subsequent to initial analysis, libraries from all sampling locations found to have average human DNA content of 8.16% or greater were enriched by bait capture^84^ for regions in the human 1240k^27^ reference dataset. Sequencing was done via a 75 bp paired-end kit on an Illumina HiSeq 4000 platform to a depth of approximately 5 million reads for initial screening and approximately 40 million reads following 1240k capture enrichment.

### Evaluation criteria

One of the most common metrics for the evaluation of molecular preservation in archaeological remains percentage of endogenous (i.e. human) aDNA recovered after sequencing. However, a high percentage of endogenous DNA on its own provides limited information on the utility of a given DNA library for downstream analysis. For example, it is important that both the proportion of human DNA relative to that of potential contaminants as well as the quantity (e.g. the number of sequences mapping to the reference as well as the as the proportion of the reference actually covered) of human DNA are high for whole genome sequencing, whereas the quantity alone is the most important criterion when using target enrichment approaches^85^. Beyond this, the integrity of the DNA molecules themselves plays an important role in the downstream mapping of sequencing data^86–88^ as well as in the authentication of ancient DNA^40,53–57^. For this reason, we integrated additional measures of data quality into our initial evaluation^89^, including the quantity of recovered human DNA, estimated DNA library complexity (in terms of both sequence duplication levels and total estimated genomic coverage), estimates of modern human DNA contamination, the ratio of nuclear to mitochondrial read recovery, average DNA fragment length, and patterns of deamination observed in reads mapping to the human reference genome. All resulting data was normalized to reflect outcomes expected from equal sequencing efforts (raw number of sequences generated prior to merging, duplicate removal, as well as length and quality filtering) across all samples where appropriate. The aim of our study was to develop a predictive model of DNA recovery based on the relative performance of each sampling location in terms of quality and quantity of recovered human DNA. We, therefore, opted not to normalize our analyses against the amount of sampling input material, despite the restricted amounts available in some locations (see Supplementary Section 2.4 for normalized analyses).

### Contamination estimates

Contamination estimates for each individual sampling location were calculated using the ANGSD^50^ software package to examine the probability of foreign X chromosome contamination in samples from male individuals using the post-capture enrichment data sets generated for eight sampling locations with human DNA recovery above 8.16%. Mitochondrial contamination estimates were generated at an individual level for all individuals using the Schmutzi^52^ software package. Multi-dimensional scaling analyses of all enriched samples was performed with the *R Statistical Software Package*^90^ using the *ggplot2* package^91^.

### Mapping

Human DNA content and sequence quality were determined by mapping reads to the hg19 human reference genome (accession number: GCF_000001405.13) using the EAGER^92^ pipeline: BWA^93^ settings: -n set at 0.1 and a mapping quality filter of q37. To assess resolution of the above pipeline in detecting ancient human DNA sequences, we created a simulated dataset based on the hg19 human reference for mapping evaluation and to act as a best-case scenario for comparative purposes. We first cut the reference sequence into fragments of average length and size distribution modelled after a representative sample (KRA001.B0102, petrous pyramid single-stranded library; see Supplementary File 1: Average and Median length). We then used the software Gargammel^94^ to artificially add a deamination pattern to the data that simulated an ancient DNA damage signal consistent with the same sample (see Supplementary File 1: Damage signals). The resulting simulated aDNA dataset was then mapped as above.

### Calculations

Percentage of human reads recovered from each sampling effort was calculated as:

1 $$\frac{{{\text{Total }}\;{\text{number }}\;{\text{of }}\;{\text{reads}}\;{\text{ mapping }}\;{\text{to }}\;{\text{reference }}\;{\text{prior}}\;{\text{ to }}\;{\text{duplicate }}\;{\text{removal}}\;{\text{ and }}\;{\text{post }}\;{\text{quality}}\;{\text{ filtering}}}}{{{\text{Total }}\;{\text{reads }}\;{\text{after }}\;{\text{merging }}\;{\text{and}}\;{\text{ filtering}}\;{\text{ for }}\;{\text{quality }}\;{\text{and }}\;{\text{length}}}}$$

The number of unique reads mapping to the human genome per million reads sequencing effort was calculated as:

2 $$\frac{{{\text{Number }}\;{\text{of }}\;{\text{reads }}\;{\text{mapping }}\;{\text{to }}\;{\text{reference }}\;{\text{after }}\;{\text{duplicate }}\;{\text{removal }}\;{\text{and }}\;{\text{quality}}\;{\text{ filtering}}}}{{{\text{Number}}\;{\text{ of }}\;{\text{reads }}\;{\text{generated}}\;{\text{ pior}}\;{\text{ to }}\;{\text{merging}}\;{\text{ or }}\;{\text{filtering}}}} \times 1,000,000$$

Total genomic coverage within a library^44^ was estimated by calculating:

3 $$\frac{{{\text{Number }}\;{\text{of }}\;{\text{DNA }}\;{\text{molecules }}\;{\text{in }}\;{\text{library}} \times {\text{Proportion }}\;{\text{of }}\;{\text{human}}\;{\text{ DNA}}\;{\text{ recovered}} \times {\text{Avg}}{.}\;{\text{ length }}\;{\text{of }}\;{\text{mapping }}\;{\text{reads}}}}{{{\text{Length }}\;{\text{of}}\;{\text{ reference }}\;{\text{genome}}}}$$

### Mixed effects modelling

All statistical analyses involving generalized linear models and mixed effects models described here were performed using the *R Statistical Software Package*^90^, where a p-value of 0.05 was considered significant. When multiple hypotheses were performed, p-values were adjusted to control for a family-wise error rate of 0.05 using the *p.adjust* function.

In all mixed effects models we considered the skeletal element to be a fixed effect with the individual as a random effect. Backward model selection was performed using ANOVA, including for testing whether random effects in the final analyses were deemed significant.

When modelling response variables with an obvious upper bound (i.e. endogenous DNA content of 100%), we implemented beta mixed effects regression as implemented in the *glmmTMB* package^95^. Optimal power transformations for theoretically unbounded response variables were performed using a Box–Cox transformation as implemented in the *MASS* package^96^.

We compared the effects of skeletal elements on response variable by inspecting the estimated marginal means in our optimal mixed effects and fixed effects models using the *emmeans* package^97^.

All visualizations of analyses included in this manuscript were produced in the R environment using the ggplot2 package^91^.

## Supplementary information

Supplementary Information 1.

Supplementary Information 2.

## Data Availability

Sequence data is available through the European Nucleotide Archive under accession number PRJ-EB36983.

## Code availability

All programs and R libraries used in this manuscript are freely and publicly available from their respective authors. All custom written R code is available by request.
