# Supplementary material for: A systematic investigation of human DNA preservation in medieval skeletons
Source: Sci Rep. 2020 Oct 26;10:18225. doi: 10.1038/s41598-020-75163-w (PMC7588426; doi:10.1038/s41598-020-75163-w)
Supplement: Supplementary file 1 — Supplementary Information 1. [file 41598_2020_75163_MOESM1_ESM.docx]

**A systematic investigation of human DNA preservation in medieval skeletons: Supplementary material**

Cody Parker^1*^, Adam B. Rohrlach^1,2^, Susanne Friederich^3^, Sarah Nagel^4^, Matthias Meyer^4^, Johannes Krause^1*^, Kirsten I. Bos^1^, Wolfgang Haak^1*^

Affiliations:

^1^Max Planck Institute for the Science of Human History, Jena, Germany

^2^ARC Centre of Excellence for Mathematical and Statistical Frontiers, The University of Adelaide, Adelaide, South Australia, Australia

^3^Landesamt für Denkmalpflege und Archäologie, Sachsen-Anhalt, Halle a. d. Saale, Germany

^4^Max Planck Institute for Evolutionary Anthropology, Leipzig, Germany

Corresponding Authors:

Cody Parker: parker@shh.mpg.de*

Wolfgang Haak: haak@shh.mpg.de*

Johannes Krause: krause@shh.mpg.de*

Adam B. Rohrlach: rohrlach@shh.mpg.de

Susanne Friederich: sfriederich@lda.stk.sachsen-anhalt.de

Sarah Nagel: [sarah_nagel@eva.mpg.de](mailto:sarah_nagel@eva.mpg.de)

Matthias Meyer: mmeyer@eva.mpg.de

Kirsten I. Bos: bos@shh.mpg.de

**1. Laboratory Processing**

**1.1 Pre-treatment**

All samples were initially cleaned with 0.01% v/v bleach to remove dirt, then rinsed with distilled water before being exposed to ultraviolet light for 30 minutes.

**1.2 Bone powder generation**

All bone powder was generated by drilling using a standard dental drill with standard drill bit on a low-speed, high-torque setting unless otherwise noted.

**1.2.1 *Pars Petrosa***

The petrous pyramid was first cut in half along the lateral line using a jeweller's saw (Figure S1b). The interior portion was then visibly examined and bone powder generated from the densest area (Figure S1a).


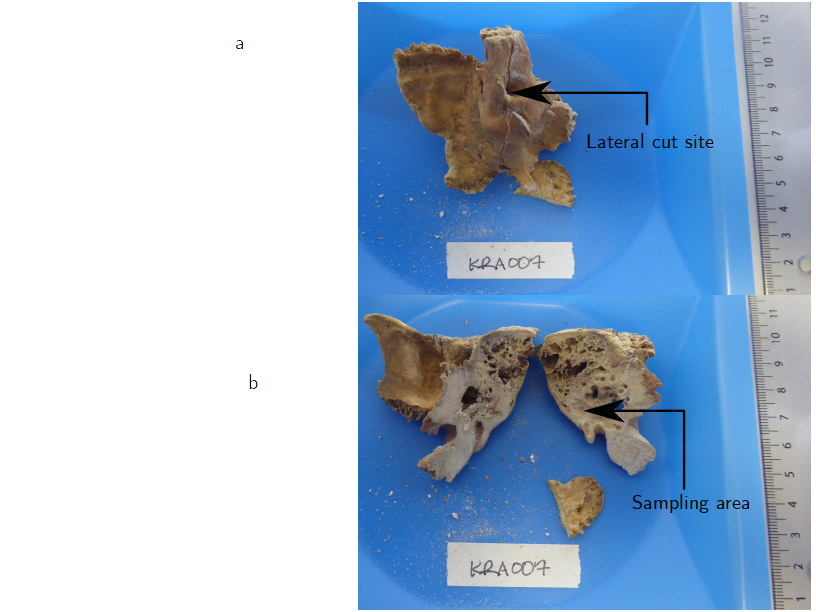


**Figure S1a-b:** Posterior view of *Pars petrosa* pre-sectioning (a) and post, showing the sectioning and subsequent drilling sites (b).

**1.2.2 Teeth**

Cementum was removed from the root portion of the tooth using a standard dental drill fitted with a circular cutting attachment. The blade of the cutting wheel was placed lightly against the root at a 20° angle (relative to the bottom of the root) on a low-speed, high-torque setting and the cementum scraped off downward (Figure S2a-b). The tooth was then bisected along the cementum-enamel junction. Powder from pulp chamber was generated using a standard dental drill bit from the first pass of the interior of the crown. Subsequent passes were used to generate bone powder from dentin (Figure S2c).

**
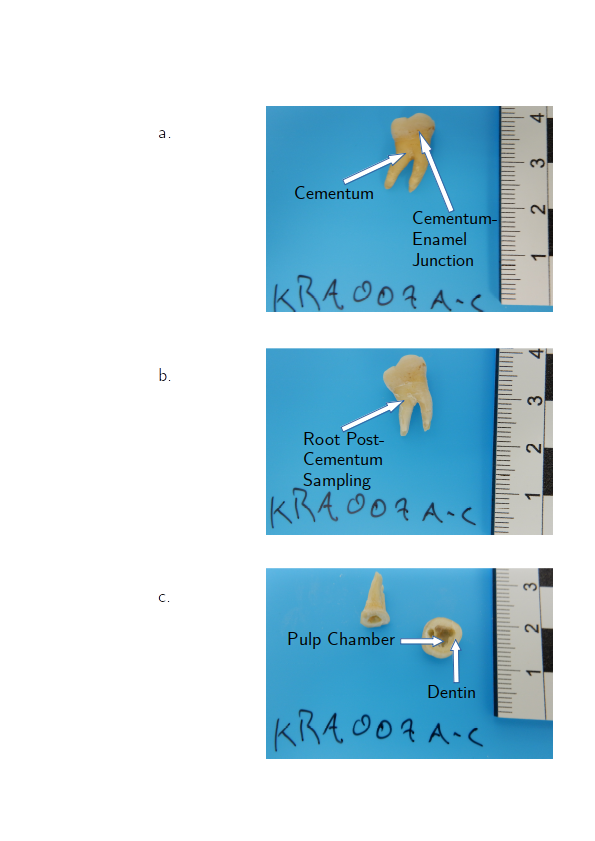
**

**Figure S2a-c:** *In situ* molar pre (a) and post (b) removal of cementum, as well as pre (b) and post (c) sectioning and drilling of the pulp chamber and underlying dentin.

**1.2.3 Clavicles**

Cortical bone powder was collected from the exterior apex of anterior sternal curve of the shaft of the clavicle, and cancellous bone powder from the interior of the acromial facet (Figure S3).


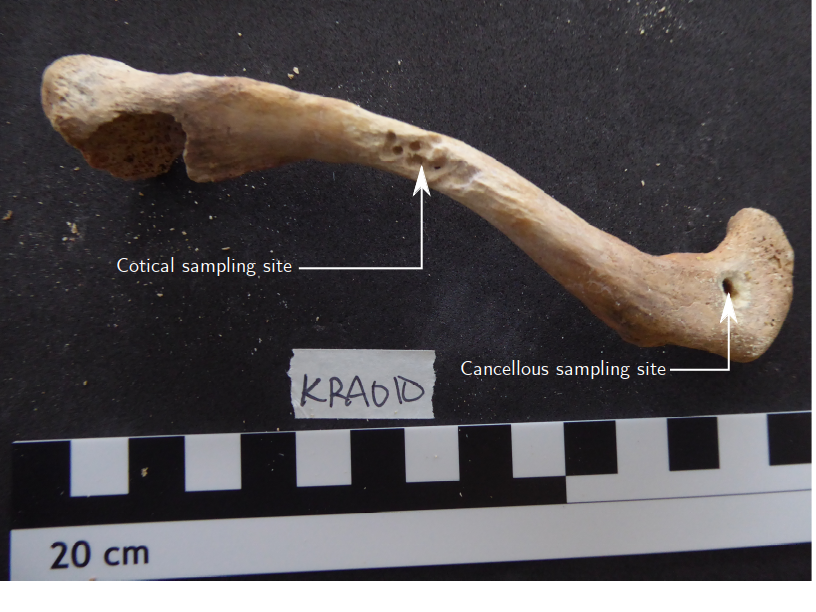


**Figure S3:** Sampling sites of the clavicle (anterior view) showing drilling locations for both cortical and cancellous tissue.

**1.2.4 Vertebrae**

Cortical bone powder was generated from the spine of the spinous process, the exterior of the vertebral body, the interior surface of the neural foramen, and the superior apex of junction of the lamellae and spinous process (superior vertebral arch). Cancellous bone powder was collected from the interior of the vertebral body (Figure S4).


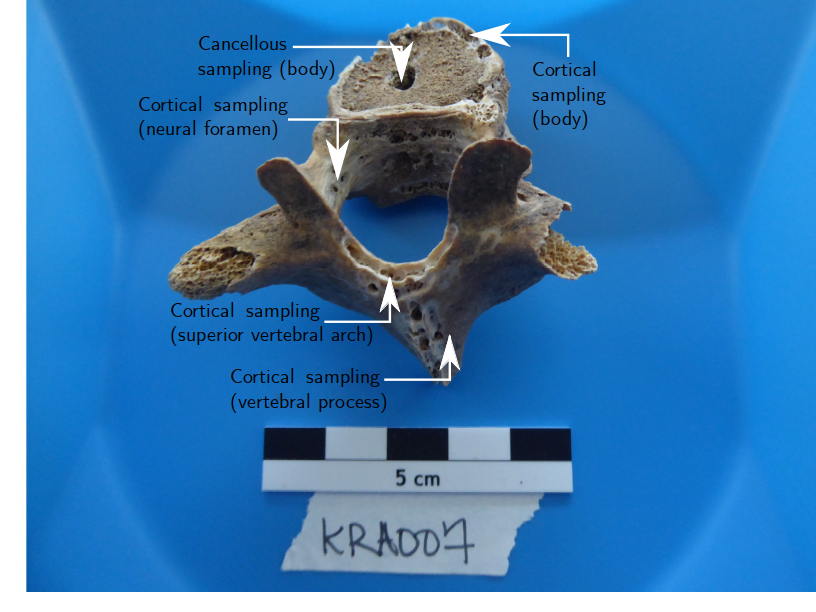


**Figure S4:** All sampling locations (post-drilling) of the thoracic vertebrae (superior view)**.**

**1.2.5 Ribs (1^st^)**

Cortical bone powder was sampled from the outer surface of the serratus anterior, cancellous powder from the interior of the costoclavicular ligament attachment site (Figure S5).


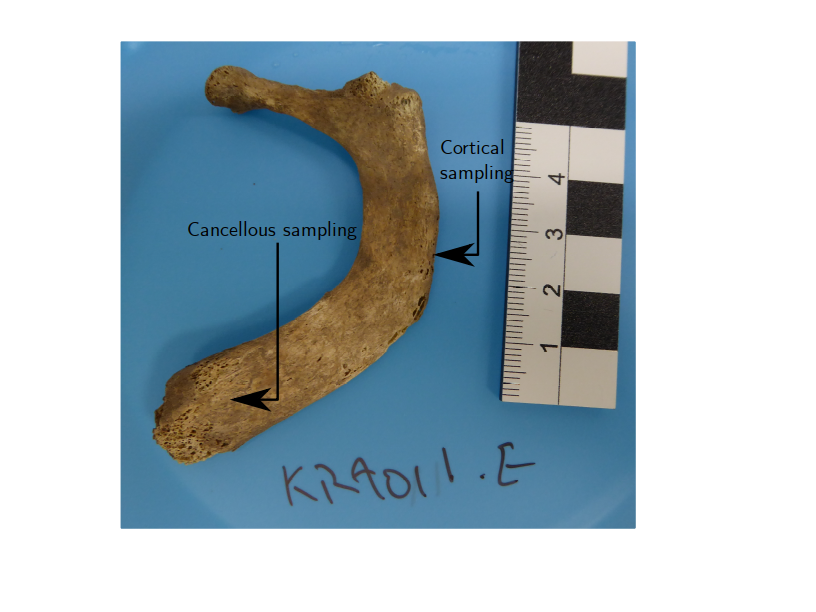


**Figure S5:** Cortical and Cancellous sampling locations on the first rib (superior surface).

**1.2.6 Metacarpals**

Cortical bone was collected from the exterior surface of the shaft, cancellous from the interior of the head (Figure S6).


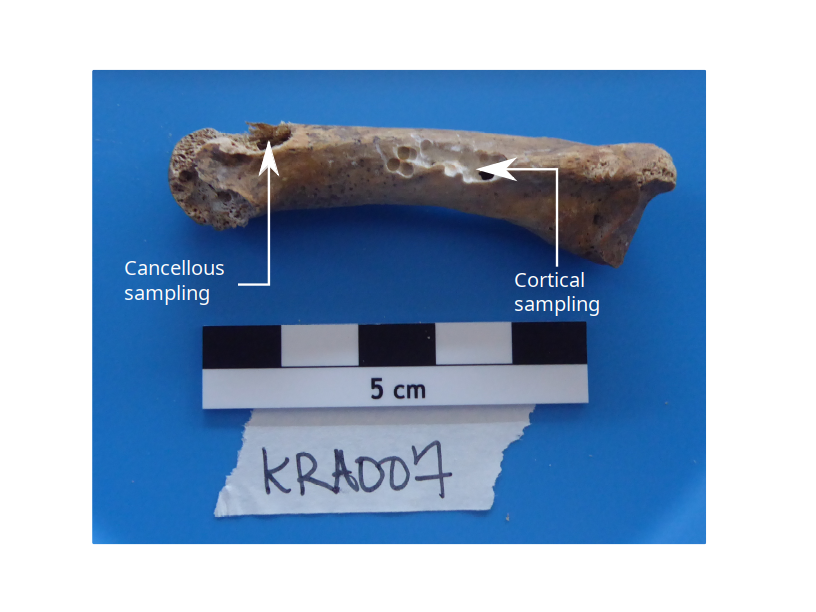


**Figure S6:** Metacarpal (palmar aspect) showing drilling locations for the collection of both cortical and cancellous tissue

**1.2.7 Distal Phalanx**

Cortical bone powder was collected from the pad and shaft of the distal phalanx, cancellous bone from the interior of the base (Figure S7).


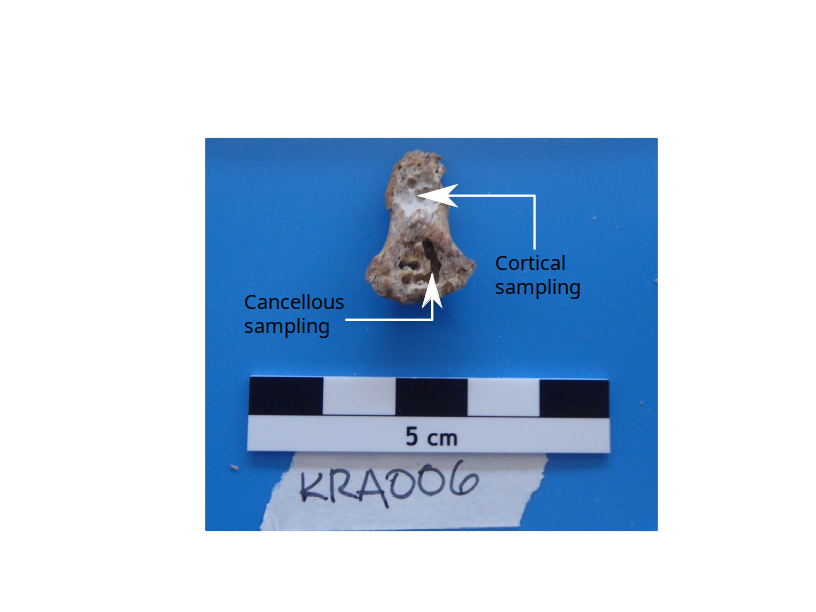


**Figure S7:** Distal phalanx (palmar aspect) showing drilling locations for the collection of both cortical and cancellous tissue.

**1.2.8 Ischial Tuberosity**

Cortical bone was collected from the exterior surface of the tuberosity, cancellous from the interior (Figure S8).


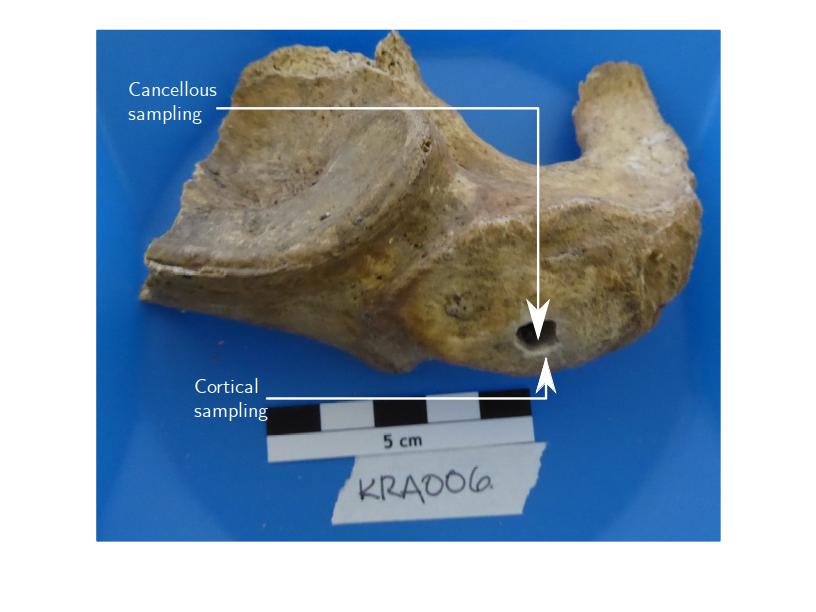


**Figure S8:** Ischial tuberosity (lateral view) showing drilling sites for the collection of both cortical and cancellous material.

**1.2.9 Femora**

Cortical bone material was collected from the shaft, just below the lesser trochanter and cancellous from the interior of the head (Figure S9).


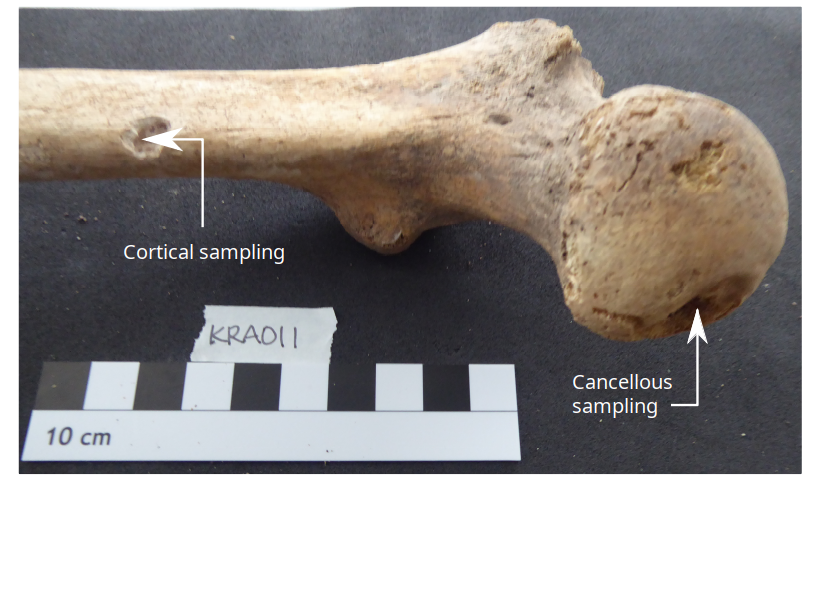


**Figure S9:** Femur (anterior view) showing drilling sites for the collection of both cortical and cancellous material.

**1.2.10 Tali**

Dense tissue was collected from the “neck” and articular surface, less compact cancellous from the interior of the medial facet (Figure S10).

**Figure S10:**
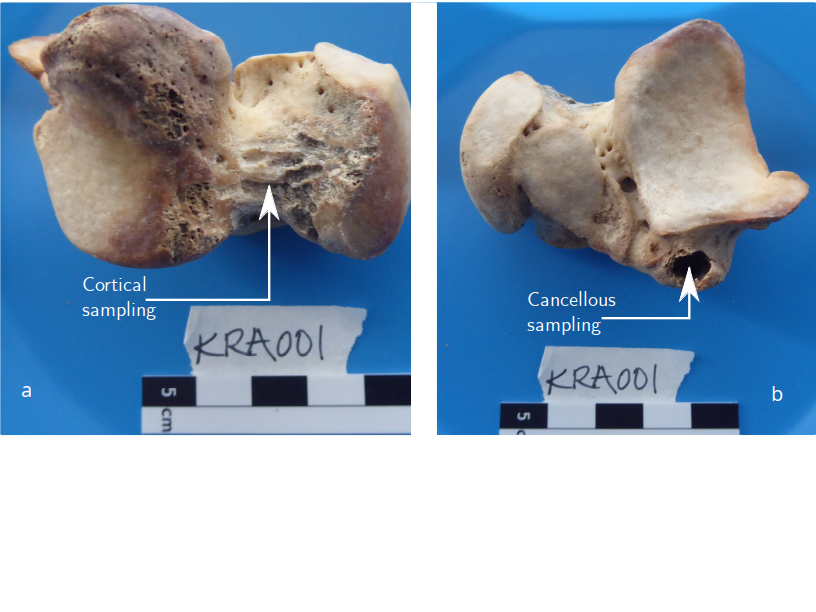
Talus showing drilling sites for the collection of both cortical (a; lateral surface and loosely packed cancellous tissue (b; inferior surface).

**1.3 DNA extraction**

One millilitre of UV-purified extraction buffer (900µl 0.5M EDTA; 75µl UV-treated, HPLC grade water; and 25µl 10mg/ml Proteinase K) was added to approximately 50mg of bone powder (where available) in a 2ml Eppendorf Biopur microcentrifuge tube and incubated (with rotation) overnight at 37°C. The mixture was then centrifuged for 2 minutes at 14000rpm and the supernatant collected and transferred to 50ml Falcon tube containing 10ml of UV-treated binding buffer (6ml 5M GuHCl, 4ml isopropanol) and 400µl 3M sodium acetate (pH: 5.2) and the contents mixed by inversion. This was then transferred into the funnel of a High Pure Viral Extract Extender Assembly and centrifuged for 8 minutes at 1500rpm. The column was then removed from the funnel and transferred to a fresh collection tube before being dry-centrifuged for 2 minutes at 14000rpm. 450µl of wash buffer (provided in the High Pure Viral Nucleic Acid Extraction Kit) was then added and the column spun at 14000rpm for 1 minute. The collection tube was then emptied and the wash repeated. The column was then dry-spun at 14000rpm for 1 minute before being transferred to a 1.5ml siliconized microcentrifuge tube for elution. Elution was done in two cycles of adding 50µl of Tris-EDTA-Tween buffer (Applichem low EDTA 1x Tris-EDTA buffer, 0.05% Tween 20), incubating at room temperature for 3 minutes, and centrifuged at 14000rpm for 1 minute, resulting in 100µl of purified aDNA extract.

**2. Supplemental Analysis**

**2.1 Expected proportion of human DNA recovered simulations**

**Table S1.** Frequency of observed rankings of skeletal elements in terms of human DNA-richness after 55,000 simulated samplings.

| **Skeletal Element** | **Frequency of observed rankings in endogenous DNA-richness (%)** | | | | | | | |
| --- | --- | --- | --- | --- | --- | --- | --- | --- |
|  | **1^st^** | **2^nd^** | **3^rd^** | **4^th^** | **5^th^** | **6^th^** | **7^th^** | **8^th^** |
| ***Pars petrosa*** | 41.87 | 22.08 | 14.03 | 9.68 | 6.40 | 4.20 | 2.07 | 0.81 |
| **Cementum** | 10.23 | 13.30 | 14.08 | 14.30 | 14.07 | 13.59 | 12.38 | 9.20 |
| **Dentin** | 6.34 | 9.03 | 10.74 | 12.50 | 13.80 | 15.16 | 16.94 | 16.62 |
| **Pulp** | 7.23 | 10.10 | 11.73 | 12.85 | 13.99 | 14.96 | 15.78 | 14.52 |
| **Vertebral Body** | 10.61 | 13.58 | 14.34 | 14.45 | 14.19 | 13.22 | 11.88 | 8.88 |
| **Superior Vertebral Arch** | 4.28 | 6.44 | 8.59 | 10.12 | 12.00 | 14.77 | 19.51 | 25.44 |
| **Distal Phalanx** | 10.65 | 13.91 | 14.43 | 14.30 | 14.02 | 13.48 | 11.92 | 8.42 |
| **Talus** | 9.93 | 12.72 | 13.21 | 12.94 | 12.67 | 11.76 | 10.67 | 8.06 |

**2.2 Transformed graphs for both estimated genomic coverage and nuclear to mitochondrial read ratio.**


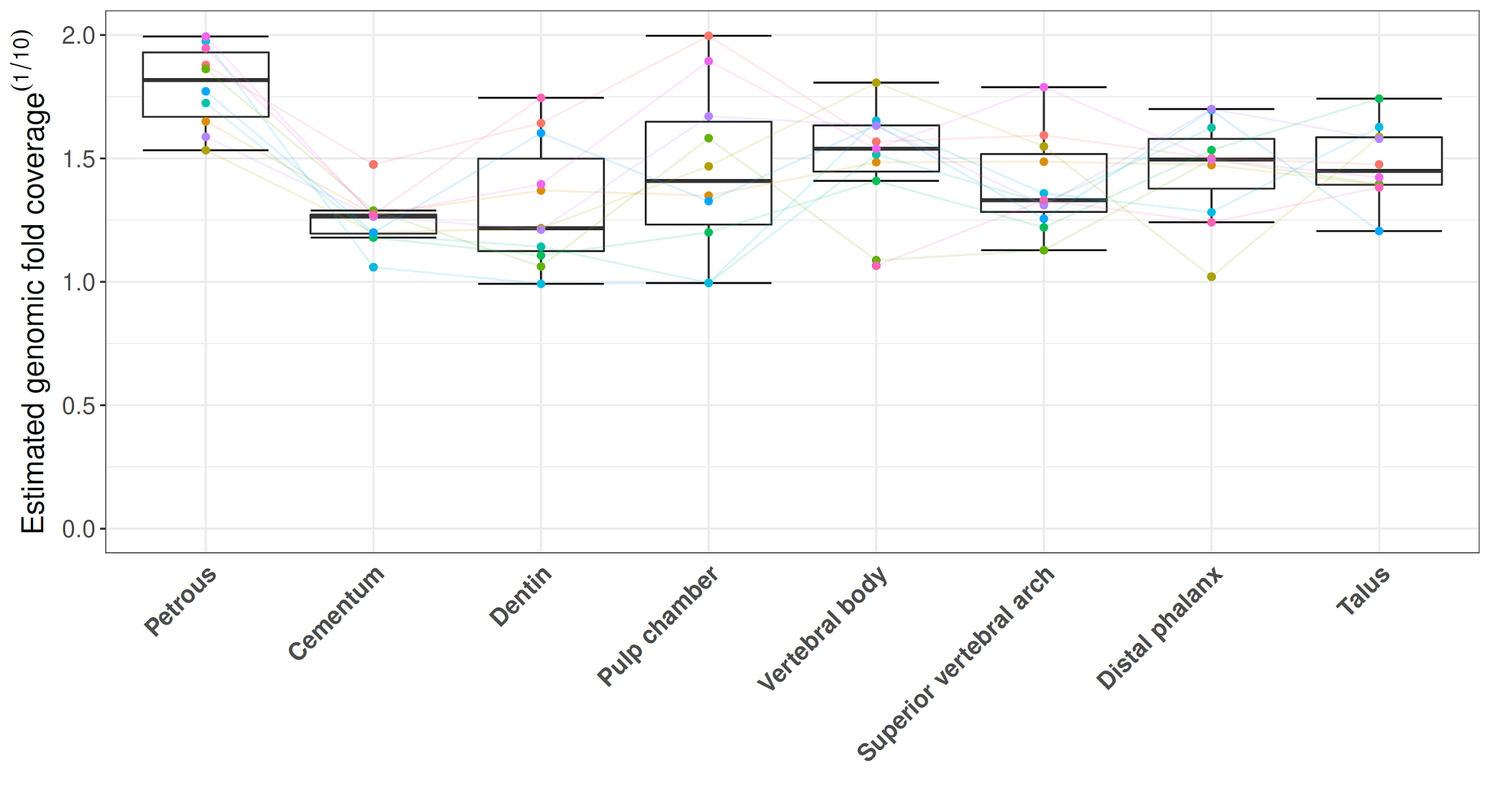


**Figure S11.** Transformed (X^0.1^) estimated genomic coverage across the eight sampling locations with average proportion of human DNA content higher than the overall mean (>8.16%). Coloured points and lines represent the genomic coverage across sampling locations within an individual.

**
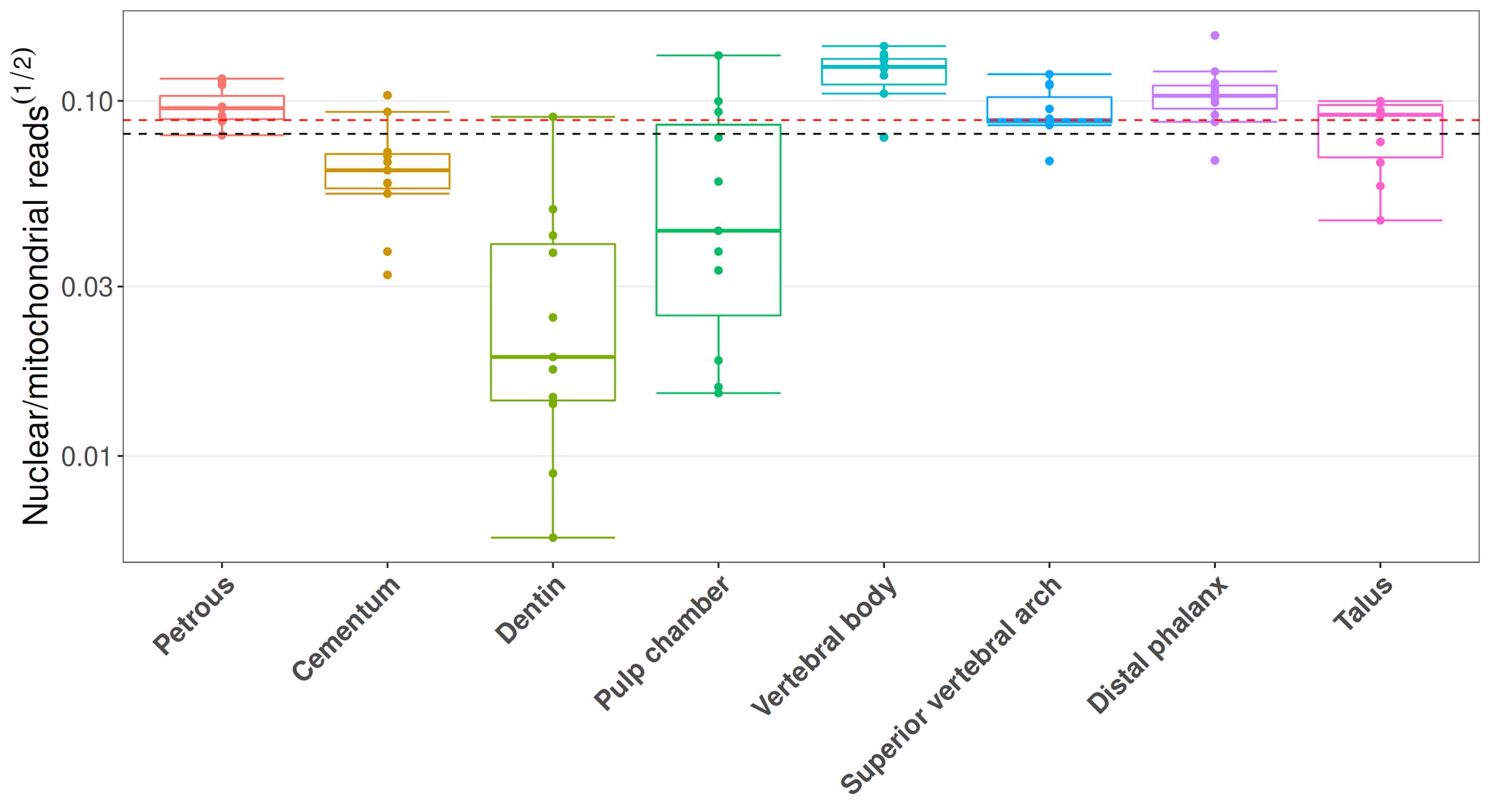
**

**Figure S12.** Transformed (X^0.5^) nuclear to mitochondrial read ratio across the eight sampling locations with average proportion of human DNA content higher than the overall mean (>8.16%). The red line represents the overall median, the black line the overall mean.

**2.3 Consistency of deamination patterns across both sampling location and individual.**

**
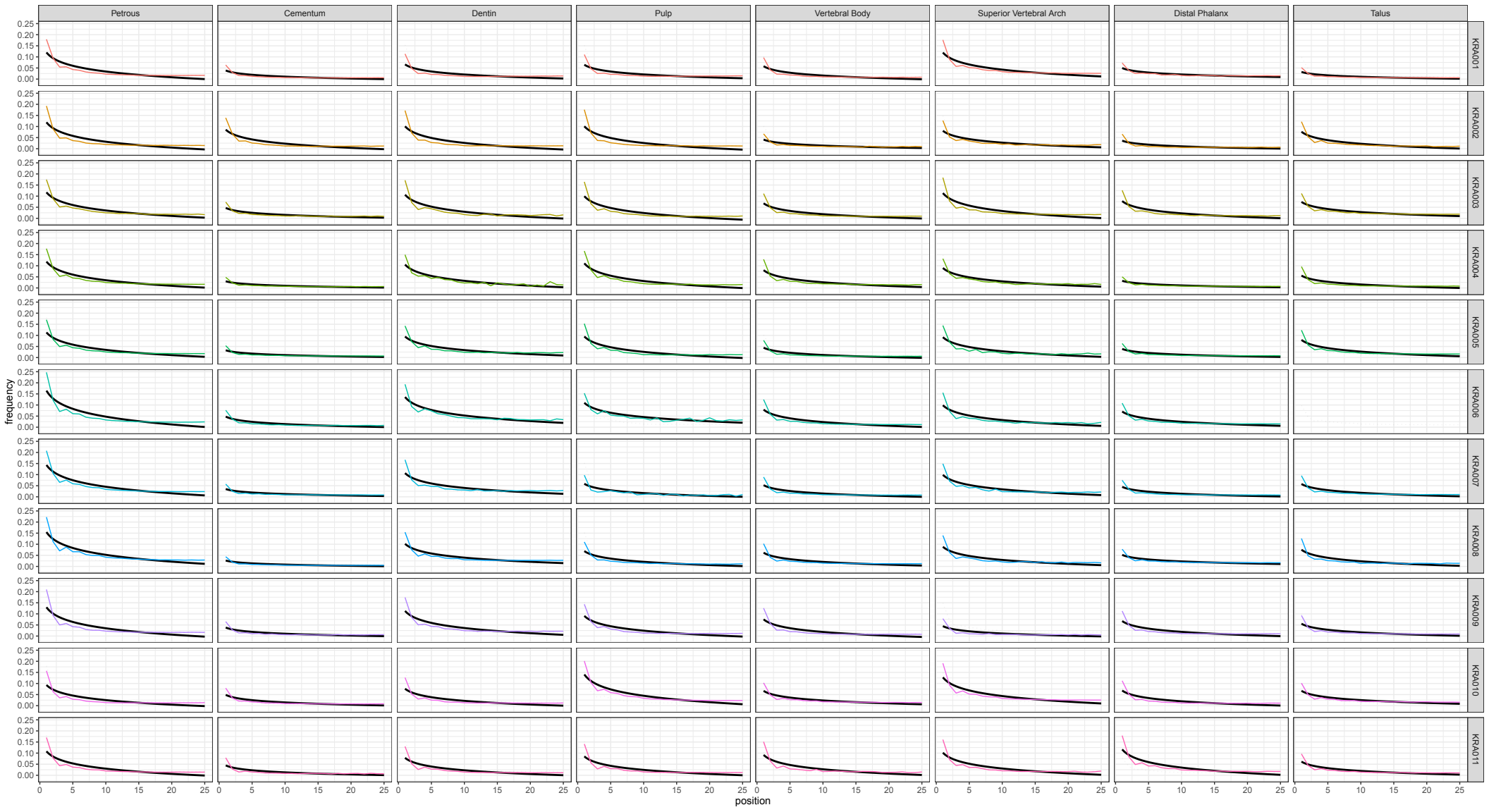
**

**Figure S13.** Deamination patterns for all eight sampling locations with higher average human DNA content than the overall mean (>8.16%) showing the consistency within each sampling location. Black lines represent lines of best fit.

**2.4 Richness per milligram input material**

The per mg richness of each individual sampling location is beyond the scope of this investigation. However, it was still noted that the cementum and the dental pulp chambers, despite yielding less starting material, were still comparable to all other sampling locations. As such, all relevant analyses were also performed after normalization of milligrams of material used in each DNA extraction.


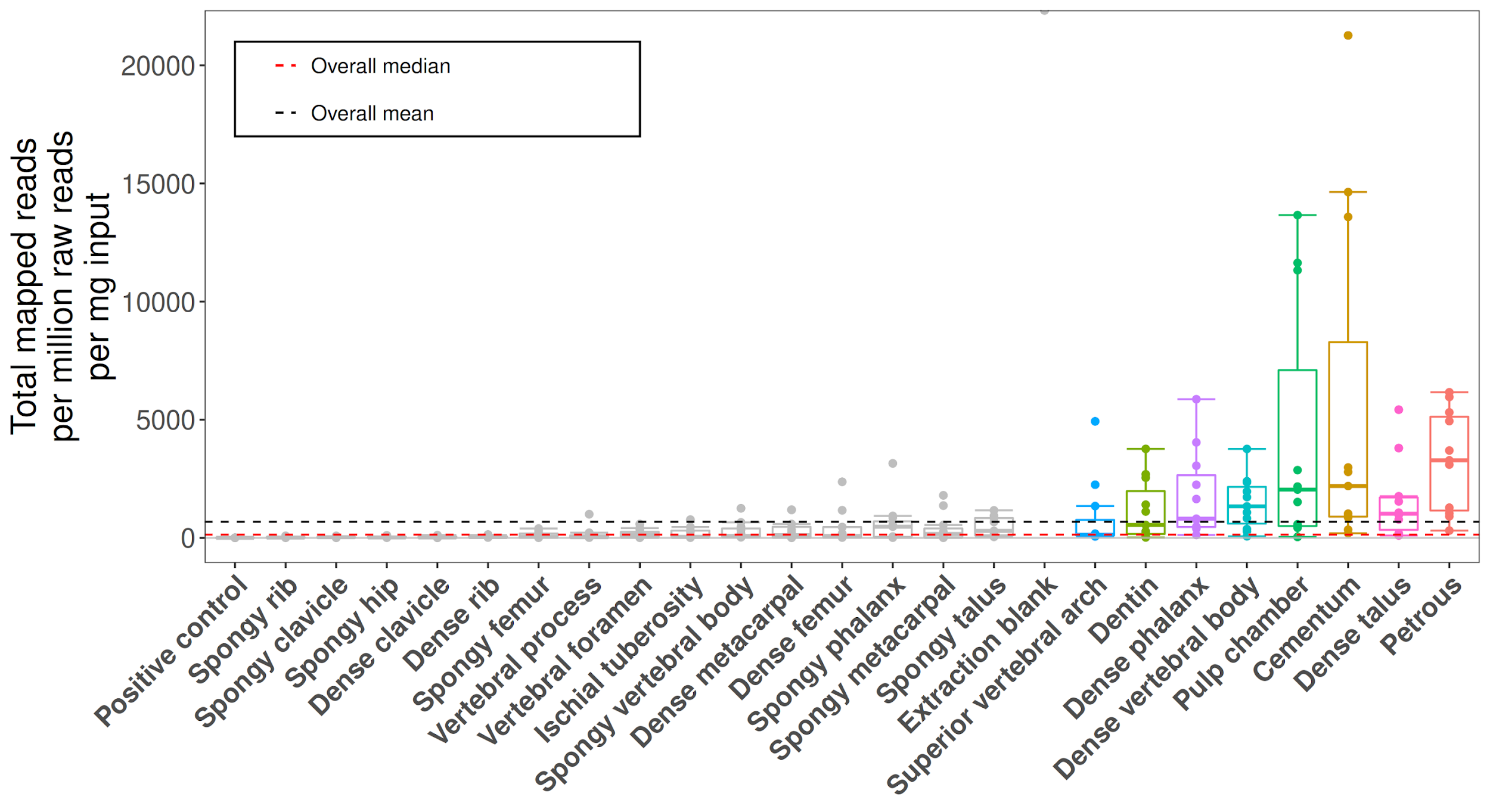


**Figure S14.** Total unique reads mapped to the hg19 human reference after normalization for starting input material and sequencing effort, showing the dental pulp chambers and cementum to be especially rich in DNA per mg. The extraction blank is included here for consistency only.


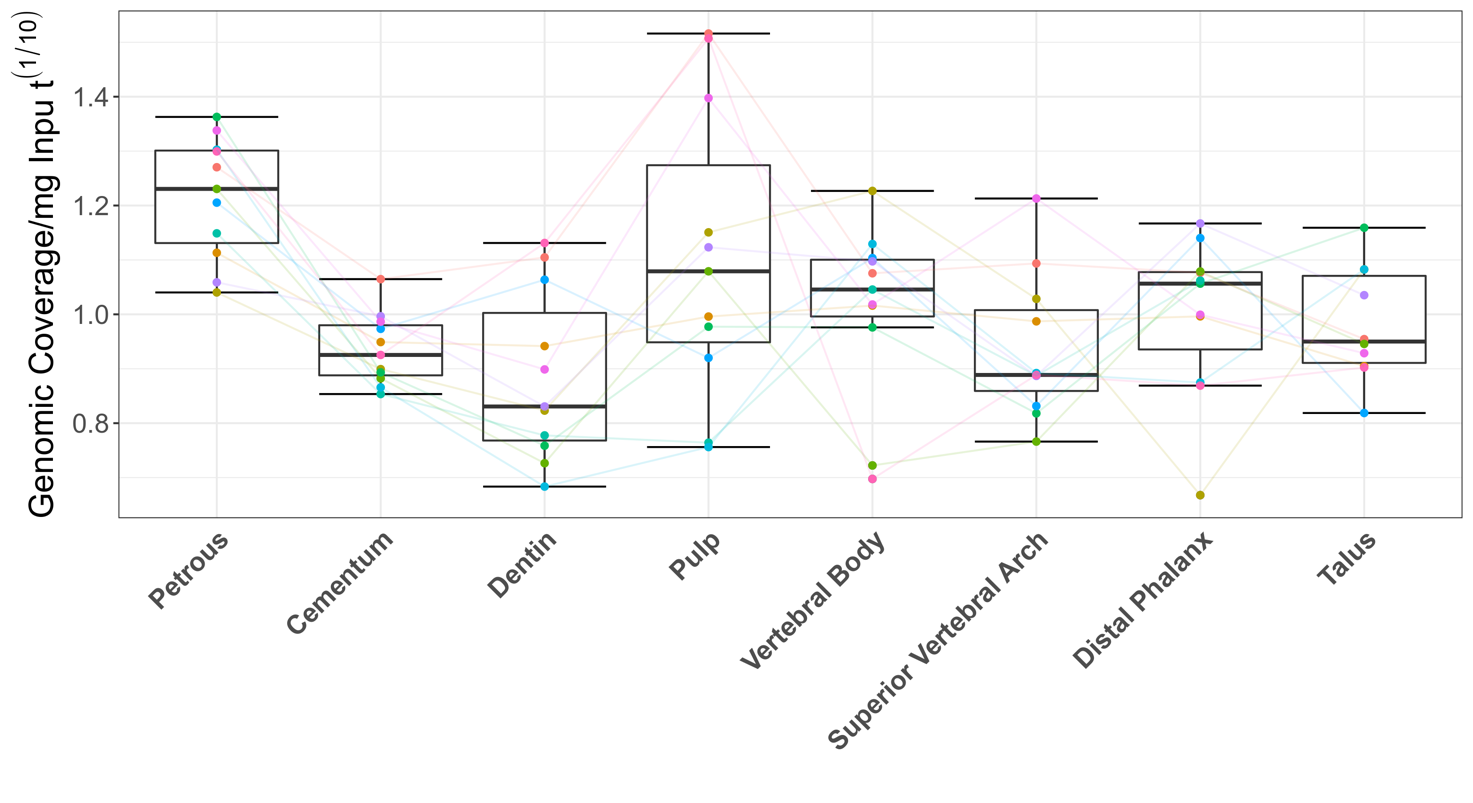


**Figure S15.** Estimated genomic coverage/mg contained within DNA libraries from each sampling location, showing the richness of the dental pulp chamber and increased richness in the cementum (to a level comparable with all other sampling locations) when input material is factored in. Coloured points and lines represent values within individuals.


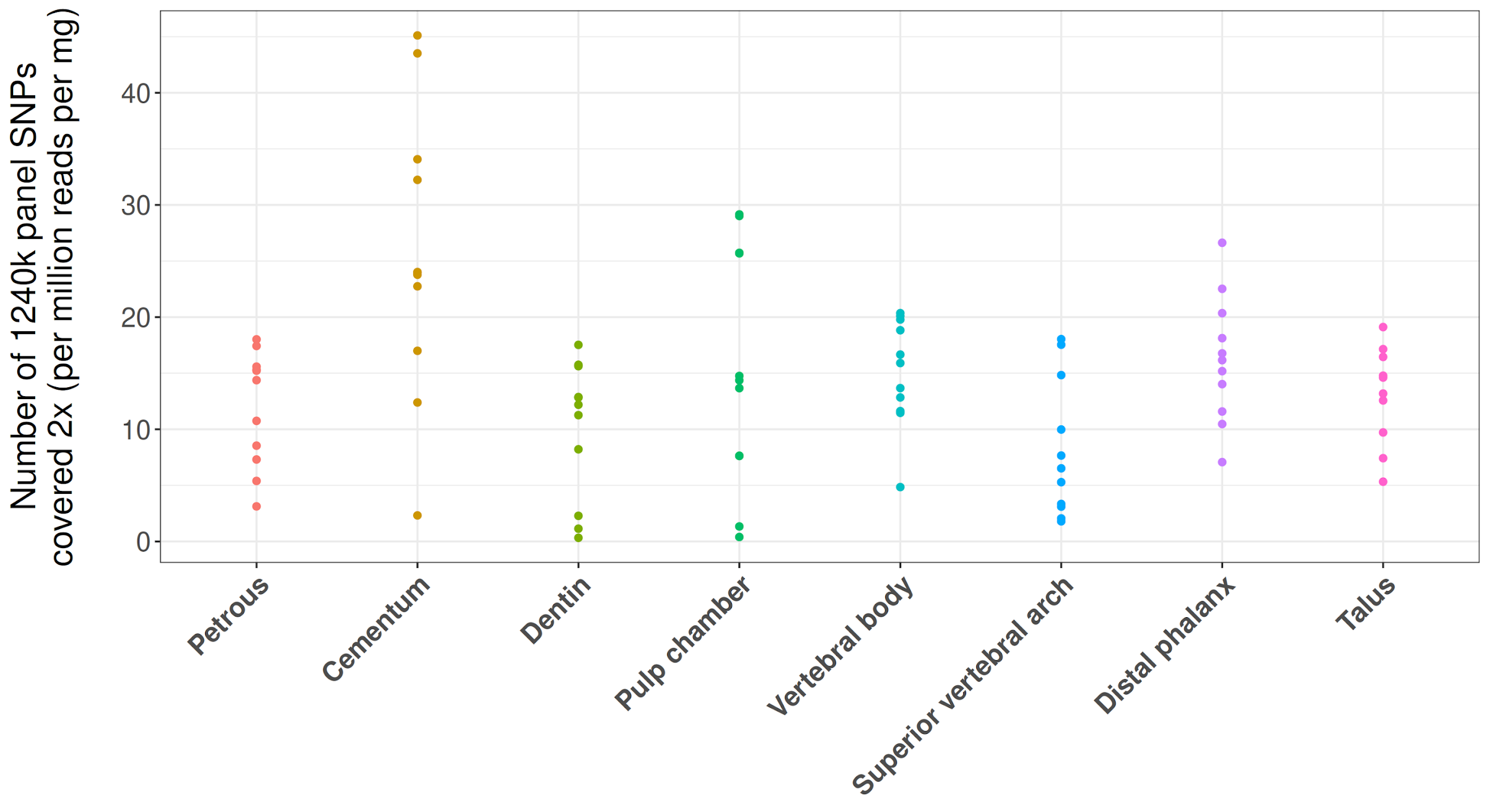


**Figure S16.** Number of 1240k panel SNPs covered at least 2x per read sequenced per mg of input material for all sampling locations, showing the increased richness of both the cementum and dental pulp chamber.

**2.5 Evaluation of epiphyseal plates found in the two juvenile individuals included in the eleven individuals used for sampling.**

Sampling was also conducted on the epiphyseal plates identified among the skeletal elements/individuals used for the main body of this study (from two individuals: in the femur, hip, and metacarpal of KRA004, in the femur and metacarpal of KRA005). As there were not enough instances of these features, they were excluded from the comparative analyses presented in the main body of this study. However, it should be noted that libraries stemming from epiphyseal plates performed well in terms of proportion of human DNA recovered, reads mapping to the human genome per million reads, and estimated genomic coverage with respect to libraries from other sampling locations from the same element in that individual (as evidenced in the corresponding Supplementary File 1). As such, further study into aDNA preservation in skeletal features such as epiphyseal plates in subadult individuals may be worthy of future consideration.

**2.6 Additional measures of contamination**

While contamination estimates derived from mitochondrial data can be useful for the evaluation of the four female individuals included in this study, they have been found to only offer similar accuracy to X chromosome contamination estimates for samples where the nuclear to mitochondrial read ratio exceeds 1:200^1^, and a such are not appropriate for all sampling locations. Additionally, as the samples in this study were not sequenced deeply enough, nor target-enriched for mitochondrial reads, the majority of sampling locations did not yield sufficient numbers of mitochondrial reads to allow the accurate calculation of mitochondrial contamination estimates. However, it was possible to combine all enriched libraries for each individual and estimate mitochondrial contamination on the individual level using the Schmutzi pipeline^2^. Here we find no detectable mitochondrial contamination (0% reported, with upper and lower bounds of 0-0.5%) in ten of the eleven individuals regardless of genetic sex. In individual KRA011 (a genetic female) we observe 12.5% potential mitochondrial contamination (upper and lower bounds of 5.5%-19.5%), but are unable to ascertain if this is a systemic issue or limited to only certain sampling locations. Of note, after visual and manual inspection of all MT reads assemblies per individual (Geneious Prime 2020.1.2; http://www.geneious.com/), we find no indication of any contaminating sources, including in the assembly of KRA011. In our experience, contamination of 12.5% should result in a clearly visible background in the read assembly. However, in the case of KRA011 deviations from the variant calls could only be observed at an average of 2.85% at C>T or G>A positions, more parsimoniously explained by residual aDNA damage. All eleven individuals produced unambiguous haplotype calls (Table S2) when applying Haplogrep2 on the consensus sequences, further adding to the fidelity of the ancient sequence data^3^.

Additionally, multi-dimensional scaling analysis was performed for all enriched libraries as a qualitative means to reinforce the ANGSD contamination estimates (Supplementary Material Figure S17), here all sampling locations from each individual clustered together, apart from the libraries corresponding to the dentin of individuals KRA003 and KRA004). It should be noted that all three of these samples are of low coverage (2,979 and 220 1240k panel SNPs covered 2x respectively, see Supplementary File 1: 1240k_SNP_Capture; covered2x_1240kpanel), making any inference of contamination based on this analysis for these samples challenging.

**Supplementary Table S2.** Individual level mitochondrial haplotype assignments

| **Individual**  **(Laboratory ID)** | **Archaeological ID**  **(Burial Nr.-Individual Nr.)** | **Mitochondrial haplotype** |
| --- | --- | --- |
| **KRA001** | 25-1a | X2x1b |
| **KRA002** | 20-2a | HV0a |
| **KRA003** | 113-6a | H73a |
| **KRA004** | 246-1a | U3a |
| **KRA005** | 276-2a | H23 |
| **KRA006** | 307-4a | H5a1a |
| **KRA007** | 377-6a | U5b1e1 |
| **KRA008** | 436-6a | T1a10 |
| **KRA009** | 566-3a | H13a1a1e |
| **KRA010** | 600-7a | H26a1 |
| **KRA011** | 632-2a | J1c4 |


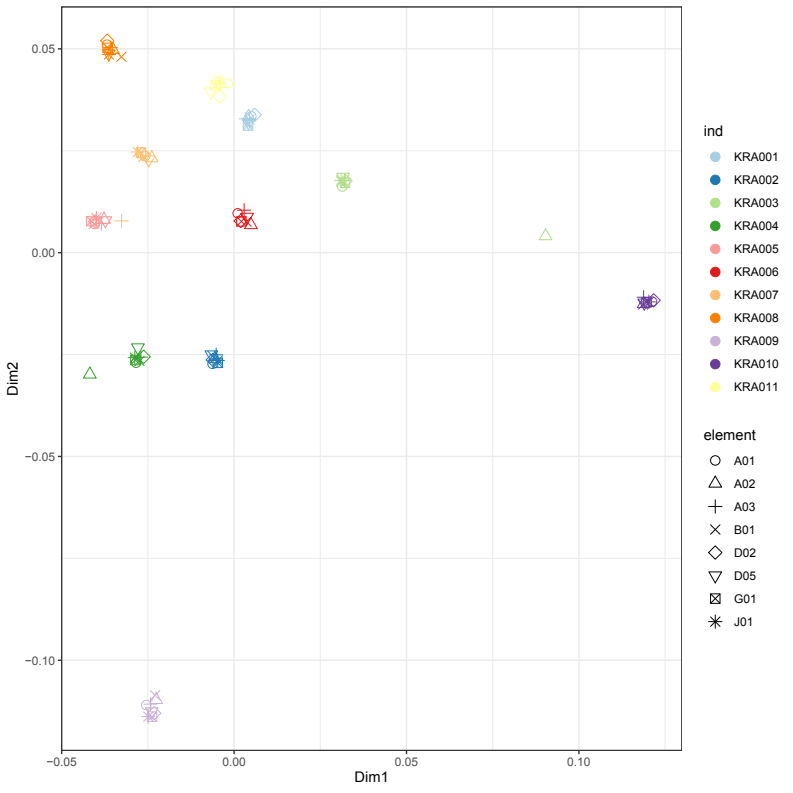


**Figure S17.** Multi-dimensional scaling plot of all 1240k enriched samples showing the consistent clustering of samples within each individual. Element codes A01, 02 and 03 refer to the cementum, dentin, and pulp chamber sampling locations (respectively); B01 to the petrous pyramid, D02 and 05 to the vertebral body and superior vertebral arch (respectively), G01 to the distal phalanx, and J01 to the talus. Samples KRA003.A02, KRA004.A02, and KRA005.A03 are low coverage samples (2,979 and 220 1240k panel SNPs covered 2x respectively).

**References**

1. Furtwängler, A. *et al.* Ratio of mitochondrial to nuclear DNA affects contamination estimates in ancient DNA analysis. *Scientific Reports* **8**, 1–8 (2018).

2. Renaud, G., Slon, V., Duggan, A. T. & Kelso, J. Schmutzi: estimation of contamination and endogenous mitochondrial consensus calling for ancient DNA. *Genome Biology* **16**, 224 (2015).

3. Weissensteiner, H. *et al.* HaploGrep 2: mitochondrial haplogroup classification in the era of high-throughput sequencing. *Nucleic Acids Res.* **44**, W58-63 (2016).
